# Computational modeling of the neural representation of object shape in the primate ventral visual system

**DOI:** 10.3389/fncom.2015.00100

**Published:** 2015-08-04

**Authors:** Akihiro Eguchi, Bedeho M. W. Mender, Benjamin D. Evans, Glyn W. Humphreys, Simon M. Stringer

**Affiliations:** ^1^Department of Experimental Psychology, Oxford Centre for Theoretical Neuroscience and Artificial Intelligence, Oxford UniversityOxford, UK; ^2^Department of Experimental Psychology, Oxford Cognitive Neuropsychology Centre, Oxford UniversityOxford, UK

**Keywords:** ventral visual pathway, neural network, trace learning, V4, TEO, shape representation, hierarchical networks

## Abstract

Neurons in successive stages of the primate ventral visual pathway encode the spatial structure of visual objects. In this paper, we investigate through computer simulation how these cell firing properties may develop through unsupervised visually-guided learning. Individual neurons in the model are shown to exploit statistical regularity and temporal continuity of the visual inputs during training to learn firing properties that are similar to neurons in V4 and TEO. Neurons in V4 encode the conformation of boundary contour elements at a particular position within an object regardless of the location of the object on the retina, while neurons in TEO integrate information from multiple boundary contour elements. This representation goes beyond mere object recognition, in which neurons simply respond to the presence of a whole object, but provides an essential foundation from which the brain is subsequently able to recognize the whole object.

## 1. Introduction

### 1.1. Hierarchical representations in the primate ventral visual pathway

Over successive stages of processing, the primate ventral visual pathway develops neurons that respond selectively to objects of increasingly complex visual form (Kobatake and Tanaka, 1994), going from simple orientated line segments in area V1 (Hubel and Wiesel, 1962) to whole objects or faces in the anterior inferotemporal cortex (TE) (Perrett et al., 1982; Tsunoda et al., 2001; Tsao et al., 2003). In addition, in higher layers of the ventral pathway, the responses of neurons to objects and faces show invariance to retinal location, size, and orientation (Tanaka et al., 1991; Rolls et al., 1992; Perrett and Oram, 1993; Rolls, 2000; Rolls and Deco, 2002). These later stages of processing carry out object recognition by integrating information from more elementary visual features represented in earlier layers (Brincat and Connor, 2004). Thus, in order to understand visual object recognition in the primate brain, we need also to understand the encoding of more elementary features in the early and middle stages of the ventral visual pathway. In particular, many theories suppose that object recognition operates through the computation of intermediate representations which reflect the spatial relations between the parts of objects (Giersch, 2001; Pasupathy and Connor, 2001; Brincat and Connor, 2004).

Experimental studies have shown that neurons in successive stages of the primate ventral visual pathway encode the spatial structure of visual objects and their parts. For example, single unit recording studies carried out by Pasupathy and Connor (2001) have shown that, within an intermediate stage of the ventral visual pathway, area V4, there are neurons that respond selectively to the shape of a local boundary element at a particular position in the frame of reference of the object. Some of these V4 neurons also maintain their response properties as an object shifts across different locations on the retina (Pasupathy and Connor, 2002). Further experimental studies have shown that neurons in the later stages of the ventral visual pathway, TEO and posterior TE, integrate information from multiple boundary contour elements (Brincat and Connor, 2004). This representation of the detailed spatial form of the separate parts of each object may provide a necessary foundation for the subsequent recognition of whole objects. That is, object selective cells at the end of the ventral visual pathway may learn to respond to unique distributed representations of object shape in earlier areas (Booth and Rolls, 1998).

### 1.2. Computer modeling study

A number of modeling studies have tried to reproduce the observed shape selective and translation invariant firing properties of neurons in area V4 (Cadieu et al., 2007; Rodríguez-Sánchez and Tsotsos, 2012). However, these past models have not utilized biologically plausible local learning rules, which use pre- and post-synaptic cell quantities to drive modification of the synaptic connections during visually-guided learning. Therefore, it still remains a challenge to understand exactly how V4 neurons develop their shape selective response properties through learning. The purpose of this paper is to provide a biologically plausible theory of this learning process. More generally, we investigate through computer simulation how the cell firing properties reported in visual areas V4, TEO, and posterior TE may develop through visually-guided learning, and thus how the primate ventral visual pathway learns to represent the spatial structure of objects.

**Figure 1:**
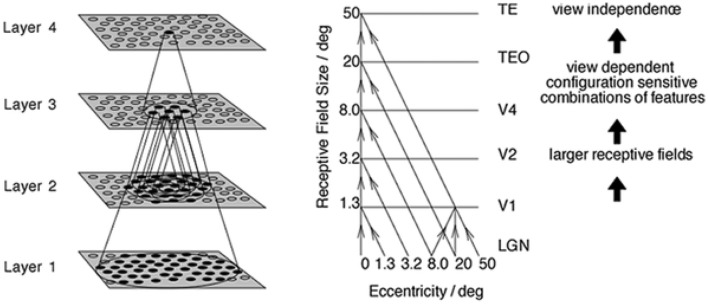
**Left:** Stylized image of the four layer VisNet architecture. **Right:** Convergence in the visual system. The diagonal lines show the convergence of feed-forward connections through successive layers of the ventral visual system leading to an increase in receptive field size from 1.3° in V1 to 50° in TE (Figures excerpted from Wallis and Rolls, 1997).

The simulation studies presented below are conducted with an established neural network model of the primate ventral visual pathway, VisNet (Wallis and Rolls, 1997), shown in Figure 1. The standard network architecture consists of a hierarchy of four competitive neural layers (Rumelhart and Zipser, 1985) corresponding to successive stages of the ventral visual pathway. The VisNet architecture is feed-forward with lateral interactions within layers. Many engineering approaches to efficiently solve similar problems extensively rely their architectures on top-down information flows, mainly for their supervised learning. However, our aim is to pin down the simplest form of core-mechanisms in intermediate vision, that is sufficient to explain a specific brain function. In fact, in other feature hierarchical neural network modeling studies, such top-down information transfer is often excluded (Olshausen et al., 1993; Riesenhuber and Poggio, 1999; Serre et al., 2005, 2007; Wallis, 2013).

The researchers involved in these last publications acknowledge the extensive presence of such back projections in the visual cortex; however, they also think the exact roles of these projections still remain a matter of debate. For example, it has been proposed that the role of these feedback pathways is to relay the interpretations of higher cortical areas to lower cortical areas in order to verify the high-level interpretation of a scene (Mumford, 1992) or to refine the tuning characteristics of lower-level cortical cells based upon the interpretations made in higher cortical areas (Tsotsos, 1993). On the other hand, numerous physiological studies have also reported that only short time spans are required for various selective responses to appear in monkey IT cells, which imply that feedback processes may not be critical for coarse, rapid recognition (Perrett et al., 1992; Hung et al., 2005; vanRullen, 2008).

We also stand on the similar point of view, and learning mechanisms implemented in the current model are a direct extension of previous papers in the field (Rumelhart and Zipser, 1985). In our paper, we have applied these established learning mechanisms to the important new problem of how the primate ventral visual system learns to represent the shapes of objects.

### 1.3. Hypothesis

In this paper, we consider how biologically plausible neuronal and synaptic learning mechanisms may be applied to the challenge of explaining (i) how neurons in V4 learn to respond selectively to the shape of localized boundary contour elements in the frame of reference of the object, (ii) how neurons in areas TEO and posterior TE learn to respond to localized combinations of boundary contour elements, and (iii) how these neurons learn to respond with translation invariance as the object is shifted through different retinal locations. In particular, we hypothesize that a biologically plausible solution may be provided by combining the statistical decoupling (Stringer et al., 2007; Stringer and Rolls, 2008) that will occur between different forms of boundary contour element over a large population of different object shapes, with the use of a temporal trace learning rule to modify synaptic weights as objects shift across different retinal locations (Wallis and Rolls, 1997; Rolls, 2000).

#### 1.3.1. Neurons learn to respond to individual boundary contour elements by exploiting statistical decoupling

In previous work, we have investigated how VisNet may learn transform invariant representations of individual objects if the network is always presented with multiple objects simultaneously during training (Stringer et al., 2007; Stringer and Rolls, 2008). We have found that if VisNet is trained on different combinations of objects on different occasions and as long as there are enough objects in the total pool of objects, this will result in statistical decoupling between any two objects. This statistical decoupling forces neurons in the higher competitive layers of VisNet to learn to respond to the individual objects, rather than the combinations of objects on which the network is actually trained.

**Figure 2:**
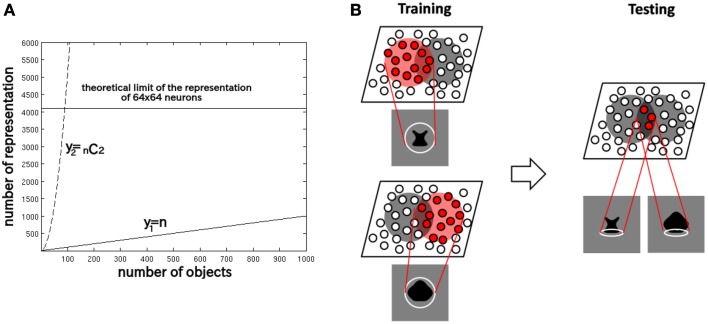
**(A)** The capacity limit of a competitive neural network forces individual output neurons to switch from representing object shapes to representing the boundary elements as the number of object shapes on which the network is trained increases. **(B)** Illustration of how the network model develops neurons that have learned to respond to individual boundary elements of 2D object shapes.

This is because a competitive neural network has a capacity limit in terms of the number of object categories that can be represented in a non-overlapping manner in the output layer. Figure 2A provides some insight into the learning mechanisms driving the formation of neurons encoding individual object. Consider the highly simplified situation where, a winner-take-all competitive network with 64 × 64 = 4096 output neurons is presented with *n* different objects, which are presented in pairs to VisNet during training. With winner-take-all competition, the network is able to develop 4096 non-overlapping output representations. Figure 2A shows how the number of individual objects, *y*_1_ = *n*, and the number of possible objects comprised of pairs of objects *y*_2_ =_*n*_ *C*_2_ = *n*(*n* − 1)∕2, rise quadratically with increasing *n*. Because of this, *y*_2_ reaches the capacity limit of the network much more quickly than *y*_1_. Therefore, for *n* > 91, individual output neurons are forced to switch from representing the objects to representing the individual objects. Although, of course, the output layer as a whole will still provide unique representations of the pairs of objects, themselves, but in a distributed, overlapping manner.

**Figure 3:**
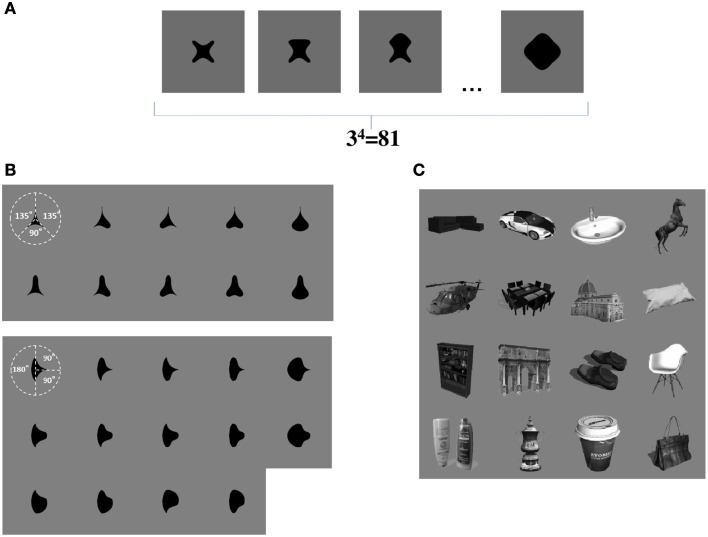
**(A)** Form of visual objects used to train and test VisNet for Study 1. Each object has a fixed number of sides (*n*), each of which has a fixed number of possible boundary conformations (*p*). **(B)** Form of visual objects used to train and test VisNet in Study 2. These visual stimuli are similar to those used in the original neurophysiological experiments of Pasupathy and Connor (2001). **(C)** Examples of some of the realistic visual objects used for training VisNet in Study 3.

We now propose that a similar learning mechanism may operate to enable the network to learn to represent the individual boundary contour elements within objects. For example, consider the simplified case shown in Figure 3A. This figure shows a set of four sided shapes, where each side has one of three possible conformations: concave, straight, or convex. Therefore, there are 4 sides × 3 side types = 12 different boundary contour elements (each defined by a unique combination of position and shape), which may be used to construct a total of 3^4^ = 81 different whole objects. We demonstrate that, when VisNet is trained on such a population of different object shapes constructed from different combinations of boundary contour elements, there is statistical decoupling between any two boundary contour elements.

Figure 2B provides an illustration of how the capacity limit forces output neurons to learn to represent individual boundary elements. Figure 2B (left) shows two different object shapes that share a boundary element at the bottom, which are presented to the network during training. Each of the two objects stimulates a subset of output neurons, and those neurons learn to represent each shape through associative learning in the feed-forward synaptic connections. The situation in the figure supposes that the subset of the neurons activated partly overlap. In this situation, the boundary element at the bottom becomes especially strongly associated with the subset of output neurons at the intersection of the two object shape representations. Figure 2B (right) shows that during testing this intersecting subset of output neurons will respond whenever the network is presented with an object shape containing the given boundary element. In this manner, without any top-down information transfer, the network should be able to develop representations of localized boundary elements. This kind of the distributed coding of 2D object shape, utilizing an alphabet of localized boundary elements, may be used to represent the shape of any object.

#### 1.3.2. Neurons develop translation invariant responses through trace learning (temporal association)

Another key property of the neurons reported by Pasupathy and Connor (2001) in area V4, and neurons reported by Brincat and Connor (2004) in areas TEO and posterior TE, is that they respond with translation invariance as an object shifts across different locations over the receptive field. The question is how these neurons might learn to respond in such a translation invariant manner?

One possible explanation is that the brain uses temporal associative learning to develop such transformation invariant representations. The theory assumes that, every now and then, a primate will make a series of fixations at different points on the same visual object before moving onto another object; much experimental work has studied the statistics of saccades and fixations across natural visual scenes (Findlay and Gilchrist, 2003). Of particular relevance is how the eyes saccade around natural visual scenes containing multiple objects. Seminal psychophysical studies of how human subjects move their gaze around pictures of natural scenes were carried out by Yarbus (1967). It was indeed evident from this work that there was a tendency for observers to shift their fixation to a number of different points on a salient object, such as a person, before moving onto the next object.

Therefore, we assume that eye movements would be sufficiently small so that the same object is always projected within the simulated receptive field when learning it. We believe this constraint is reasonable to simulate recent physiological findings. For example, Li and DiCarlo (2008) conducted a study where monkeys are trained to track an object on a screen where a object with identity A is originally placed on the one of two possible retinal positions (+3° or −3°) and later shifted to the center (0°). In the experimental condition, the identity of the object is swapped from A to B when it is shifted to the center, and the eyes saccade to it. As a results, individual neurons in primate IT that are originally translation-invariantly selective to identity A start to respond also to object with identity B only at the specific retinal location. This finding does not exclude the possibility of the temporal association learning which may occur at larger eye movement; however, it provided a reasonable evidence for the translation invariance learning mechanism within IT (Isik et al., 2012).

Accordingly, our proposed solution is *temporal trace learning* (Foldiak, 1991; Wallis and Rolls, 1997; Rolls and Milward, 2000). An example of such a learning rule is given in Section 2. If the eyes shift about a visual scene more rapidly than the objects change within the scene, then the images of an object in different locations on the retina will tend to be clustered together in time. In this case, a trace learning rule will encourage neurons in higher layers to learn to respond with translation invariance to specific objects or features across different retinal locations.

This rule is biologically plausible in terms of the way it utilizes only locally available biological quantities, that is, the present and recent activities of the pre- and post-synaptic neurons, respectively. Also, it has been shown that this type of temporal associative learning arises naturally within biophysically realistic spiking neural networks when longer time constants for synaptic conductance are introduced (Evans and Stringer, 2012).

Our past research has shown that this trace learning rule may be combined with the mechanism of statistical decoupling described above to produce translation invariant representations of statistically independent visual objects (Stringer et al., 2007; Stringer and Rolls, 2008). We hypothesize that the same trace learning rule could encourage neurons representing boundary contour elements to respond with translation invariance across different retinal locations.

### 1.4. Overview of simulation studies carried out in this paper

Study 1 provides a proof-of-principle analysis. VisNet was trained on artificial visual objects similar to those shown in Figure 3A. These carefully constructed objects allowed us to explore how the statistical decoupling between different boundary contour elements influences the neuronal firing properties that develop during learning. We also showed how the capacity of the network to represent many different boundary contour conformations can be increased by introducing a Self-Organizing Map (SOM) architecture within each layer. Finally, we used the same artificial visual stimuli to confirm that trace learning can produce neurons that respond to individual boundary contour elements with translation invariance across different retinal locations.

In Study 2, the sets of visual stimuli presented to VisNet during training and testing were similar to those used in the original physiological experiments of Pasupathy and Connor (2001). Examples are shown in Figure 3B. This allowed for a direct comparison between the performance of the VisNet model and real neurons recorded in area V4 of the primate ventral visual pathway.

In Study 3, we trained VisNet on a large number of realistic visual objects with different boundary shapes. A sample of these objects is shown in Figure 3C. This generated a more realistic and demanding test of the underlying theory.

## 2. Materials and methods

### 2.1. Hierarchical neural network architecture of the model

VisNet is a hierarchical neural network model of the primate ventral visual pathway, which was originally developed by Wallis and Rolls (1997). The standard network architecture is shown in Figure 1. It is based on the following: (i) a series of hierarchical competitive layers with local graded lateral inhibition. (ii) Convergent connections to each neuron from a topologically corresponding region of the preceding layer. (iii) Synaptic plasticity based on a biologically-plausible local learning rule such as the Hebb rule or trace rule, which are explained in Section 2.4.

In past work, the hierarchical series of four neuronal layers of VisNet have been related to the following successive stages of processing in the ventral visual pathway: V2, V4, the posterior inferior temporal cortex, and the anterior inferior temporal cortex. In this paper, we model for the first time neuronal response properties observed within a series of intermediate layers. Due to the relatively course-grained four-layer architecture of VisNet, we do not wish to emphasize a specific correspondence between the layers of VisNet and particular stages of the ventral pathway. However, as our main focus was on the neuronal properties reported in V4 and TEO, we mostly focused on the first three layers of VisNet.

In VisNet, the forward connections to individual cells are derived from a topologically corresponding region of the preceding layer, using a Gaussian distribution of connection probabilities. These distributions are defined by a radius which contained approximately 67% of the connections from the preceding layer. The values employed in the current studies are given in Table 1, which have been proposed to be realistic in Wallis and Rolls (1997). However, to deal with more complex images, the size of the layer was extended to 128 × 128 neurons from 32 × 32 neurons. The gradual increase in the receptive field of cells in successive layers reflects the known physiology of the primate ventral visual pathway (Pettet and Gilbert, 1992; Pasupathy, 2006; Freeman and Simoncelli, 2011).

**Table 1:** **VisNet parameters**.

**Layer**	**Dimensions**	**Number of connections**	**Radius**
Layer 4	128 × 128	100	12
Layer 3	128 × 128	100	9
Layer 2	128 × 128	100	6
Layer 1	128 × 128	201	6
Retina	256 × 256 × 16		

### 2.2. Pre-processing of the visual input by Gabor filters

Before the visual images are presented to VisNet's input layer 1, they are pre-processed by a set of input filters that accord with the general tuning profiles of simple cells in V1. The filters provide a unique pattern of filter outputs for each transform of each visual object, which is passed through to the first layer of VisNet. In this paper, the input filters were matching the firing properties of V1 simple cells, which respond to local oriented bars and edges within the visual field (Jones and Palmer, 1987; Cumming and Parker, 1999). The input filters used are computed by the following equations (Daugman, 1985):

(1) $$\begin{array}{l} g\left(x,y,\lambda ,\sigma ,\theta ,\psi ,\gamma \right)=\exp\left(-\frac{{x{}^{\prime }}^{2}+\gamma^{2}{y{}^{\prime }}^{2}}{2\sigma^{2}}\right)\cos\left(2\pi \frac{x^{\prime }}{\lambda }+\psi \right) \end{array}$$

with the following definitions:

(2) $$\begin{array}{l} \begin{array}{c} x^{\prime }=x\cos\theta +y\sin\theta \\ y^{\prime }=-x\sin\theta +y\cos\theta \end{array} \end{array}$$

where *x* and *y* specify the position of a light impulse in the visual field (Petkov and Kruizinga, 1997). The parameter λ is the wavelength, σ is the standard deviation which is a function of λ and spatial bandwidth *b*, θ defines the orientation of the feature, ψ defines the phase offset, and γ sets the aspect ratio. In each experiment, an array of Gabor filters is generated at each of 256 × 256 retinal locations with the parameters given in Table 2.

**Table 2:** **Parameters for Gabor input filters**.

**Parameter (Symbol)**	**Values**
Wavelength(λ)	2
Spatial bandwidth (*b*)	1.5 octaves
Orientation(θ)	0, π∕4, π∕2, 3π∕4
Phase shift (ψ)	0: white on black bar
	π: black on white bar
Aspect ratio (γ)	0.5

The outputs of the Gabor filters are passed to the neurons in layer 1 of VisNet according to the synaptic connectivity given in Table 1. Each layer 1 neuron received connections from 201 randomly chosen Gabor filters localized within a topologically corresponding region of the retina. In the original VisNet model (Wallis and Rolls, 1997), the input filters were tuned to the four different spatial wavelengths 2, 4, 8, and 16 pixels. The shortest wavelength filters provided the highest resolution information about the image. The neurons in the first layer of VisNet were thus assigned most of their afferent inputs from the shortest wavelength filters. In the current simulations reported here, the model used inputs from only the shortest wavelength filters, which was found to be sufficient to represent the simple visual objects. For consistency with past VisNet simulations, each neuron in the first layer of VisNet received afferent connections from 201 of the short wavelength filters.

### 2.3. Calculation of cell activations within the network

Within each of the neural layers 1–4 of the network, the activation *h*_*i*_ of each neuron *i* was set equal to a linear sum of the inputs *y*_*j*_ from afferent neurons *j* in the preceding layer weighted by the synaptic weights *w*_*ij*_. That is,

(3) $$\begin{array}{l} h_{i}=\underset{j}{\sum }w_{ij}y_{j} \end{array}$$

where *y*_*j*_ is the firing rate of neuron *j*, and *w*_*ij*_ is the strength of the synapse from neuron *j* to neuron *i*.

### 2.4. Lateral interaction between neurons within each layer

In the simulations reported below, the lateral interaction between the neurons within each neuronal layer was implemented in one of two different ways. The simplest approach was to implement a competitive network architecture (Rolls and Treves, 1998), in which neurons inhibited all of their neighbors. However, in some simulations we also implemented a more complex SOM architecture (Kohonen, 2000), which included both short range excitation and longer range inhibition between neurons (i.e., a “Mexican hat” connectivity). A SOM architecture leads to a map-like arrangement of neuronal response characteristics across a layer after training, with nearby cells responding to similar inputs. In particular, we investigated the hypothesis that the SOM architecture could increase the capacity of the network by enabling neurons in the higher layers to discriminate between more boundary contour shapes. Parameters shown in Tables 3, 4 were selected based on those that previously optimized performance (Rolls and Milward, 2000; Tromans et al., 2011).

**Table 3:** **Lateral inhibition parameters for the competitive network architecture**.

**Layer**	**1**	**2**	**3**	**4**
Radius (σ)	1.38	2.7	4.0	6.0
Contrast (δ)	1.5	1.5	1.6	1.4

**Table 4:** **SOM parameters**.

**Layer**	**1**	**2**	**3**	**4**
Excitatory radius (σ_*E*_)	1.4	1.1	0.8	1.2
Excitatory contrast (δ_*E*_)	5.35	33.15	117.57	120.12
Inhibitory radius (σ_*I*_)	2.76	5.4	8.0	12.0
Inhibitory contrast (δ_*I*_)	1.5	1.5	1.6	1.4

#### 2.4.1. Competitive network architecture

The original VisNet model implemented a competitive network within each layer. Within each layer, competition was graded rather than winner-take-all. To implement lateral competition, the activations *h*_*i*_ of neurons within a layer were convolved with a spatial filter, *I*_*ab*_, where δ controlled the contrast and σ controlled the width, and *a* and *b* indexed the distance away from the center of the filter:

(4) $$I_{a,b}=\{\begin{array}{cc} -\delta \exp(-\frac{a^{2}+b^{2}}{\sigma^{2}}) & a\neq 0\;or\;b\neq 0\\ 1-\sum_{a\neq 0,b\neq 0}I_{a,b} & a=0\;and\;b=0 \end{array}$$

The lateral inhibition parameters for the competitive network architecture are given in Table 3.

#### 2.4.2. Self-organizing map

In this paper, we have also run simulations with a SOM (von der Malsburg, 1973; Kohonen, 1982) implemented within each layer. In the case of the SOM architecture, short-range excitation and long-range inhibition are combined to form a Mexican-hat spatial profile and is constructed as a difference of two Gaussians as follows:

(5) $$\begin{array}{l} I_{a,b}=-\delta_{I}\exp\left(-\frac{a^{2}+b^{2}}{\sigma_{I}^{2}}\right)+\delta_{E}\exp\left(-\frac{a^{2}+b^{2}}{\sigma_{E}^{2}}\right) \end{array}$$

To implement the SOM, the activations *h*_*i*_ of neurons within a layer were convolved with a spatial filter, *I*_*ab*_, where δ_*I*_ controlled the inhibitory contrast and δ_*E*_ controlled the excitatory contrast. The width of the inhibitory radius was controlled by σ_*I*_ and the width of the excitatory radius by σ_*E*_. The parameters *a* and *b* indexed the distance away from the center of the filter. The lateral inhibition and excitation parameters used in the SOM architecture are given in Table 4.

### 2.5. Contrast enhancement of neuronal firing rates within each layer

Next, the contrast between the activities of neurons with each layer was enhanced by passing the activations of the neurons through a sigmoid transfer function (Rolls and Treves, 1998) as follows:

(6) $$\begin{array}{l} y=f^{sigmoid}\left(r\right)=\frac{1}{1+\exp\left(-2\beta \left(r-\alpha \right)\right)} \end{array}$$

where *r* is the activation after applying the lateral competition or SOM filter, *y* is the firing rate after contrast enhancement, and α and β are the sigmoid threshold and slope, respectively. The parameters α and β are constant within each layer, although α is adjusted within each layer of neurons to control the sparseness of the firing rates. For example, to set the sparseness to 4%, the threshold is set to the value of the 96th percentile point of the activations within the layer. The parameters for the sigmoid activation function are shown in Table 5. These are the standard parameter values that have been used in past VisNet studies (Stringer et al., 2006, 2007; Stringer and Rolls, 2008).

**Table 5:** **Parameters for Sigmoid activation function**.

**Layer**	**1**	**2**	**3**	**4**
Percentile	99.2	98	88	91
Slope (β)	190	40	75	26

### 2.6. Training the network: visually-guided learning of synaptic weights

The outputs of the Gabor filters were passed to layer 1 of VisNet. Activity was then propagated sequentially through layers 2 to 4 using the same mechanisms at each layer. During training with visual objects, the strengths of the feed-forward synaptic connections between successive neuronal layers are modified by local learning rules, where the change in the strength of a synapse depends on the current or recent activities of the pre- and post-synaptic neurons. Two such learning rules were implemented with different learning properties.

#### 2.6.1. The Hebb learning rule

One simple well-known learning rule is the Hebb rule:

(7) $$\begin{array}{l} \delta w_{ij}=kr_{i}^{\tau }r_{j}^{\tau } \end{array}$$

where δ*w*_*ij*_ is the change of synaptic weight *w*_*ij*_ from pre-synaptic neuron *j* to post-synaptic neuron *i*, $r_{i}^{\tau }$ is the firing rate of post-synaptic neuron *i* at timestep τ, $r_{j}^{\tau }$ is the firing rate of pre-synaptic neuron *j* at timestep τ, and *k* is the learning rate constant.

#### 2.6.2. The trace learning rule

An alternative learning rule that, in addition to producing neurons that respond to individual contour elements, can also drive the development of translation invariant neuronal responses is the trace learning rule (Foldiak, 1991; Wallis and Rolls, 1997), which incorporates a memory trace of recent neuronal activity:

(8) $$\begin{array}{l} \delta w_{ij}=k{\bar{r}}_{i}^{\tau -1}r_{j}^{\tau } \end{array}$$

where ${\bar{r}}_{i}^{\tau }$ is the trace value of the firing rate of post-synaptic neuron *i* at timestep τ. The trace term is updated at each timestep according to

(9) $$\begin{array}{l} {\bar{r}}_{i}^{\tau }=\left(1-\eta \right)r_{i}^{\tau }+\eta {\bar{r}}_{i}^{\tau -1} \end{array}$$

where η may be set anywhere in the interval [0, 1], and for the simulations described below, η was set to 0.8. The effect of this learning rule is to encourage neurons to learn to respond to visual input patterns that tend to occur close together in time. If the eyes shift about a visual scene containing a static object, then the trace learning rule will tend to bind together successive images corresponding to that object in different retinal locations.

In our simulations, natural eye movements are simulated implicitly during training by shifting each visual object in turn across a number of retinal locations. That is, to simulate natural rapid eye movements during visual inspection of each object, the visual object itself is shifted across the retina. After an object shifted through all of the retinal locations, the next object was presented across the same locations.

To prevent the same few neurons always winning the competition, the synaptic weight vector ***w***_*i*_ of each neuron *i* is renormalized to unit length after each learning update for each training pattern by setting

(10) $$w_{i}=\frac{w_{i}}{\left||w_{i}|\right|}$$

where ||**w**_*i*_|| is the length of the vector **w**_*i*_ given by

(11) $$||w_{i}||=\sqrt{\sum_{j}w_{ij}^{2}}$$

### 2.7. Testing the network

After the synaptic weights were established by training the network on a set of visual objects, the learned response properties of neurons through successive layers were tested. This was done by presenting visual objects constructed from a pool of different boundary contour elements, with the objects being similar or different to those used during training. A number of tests are applied to the recorded neuronal responses, including information theory, which are described below. We also analyzed the learned response properties of an output cell by plotting the subset of input Gabor filters with the strongest feed-forward connections to that output cell after training.

### 2.8. Information analysis

To quantify the performance in transformation invariance learning with VisNet, the techniques of Shannon's information theory have previously been used (Rolls and Treves, 1998). In particular, a single cell information measure was applied to analyse the responses of individual cells. In order to keep the notation consistent with past publications (Rolls et al., 1997; Rolls and Milward, 2000), we have here denoted the neuronal firing rates by *r*.

To be informative in the context of this study, the responses of a given neuron (*r*) should be specific to a particular contour that appears at a particular side (*s*), and independent of the remaining global form of the object or retinal location. The amount of stimulus-specific information that a certain cell transmits is calculated from the following formula with details given by Rolls and Milward (2000).

(12) $$\begin{array}{l} I\left(s,\vec{R}\right)=\underset{r\in \vec{R}}{\sum }P\left(r|s\right)\log_{2}\frac{P\left(r|s\right)}{P\left(r\right)} \end{array}$$

Here *s* is a particular stimulus (i.e., a specific contour, at a specific side) and $\vec{R}$ is the set of responses of the cell to the set of objects that contain the contour at that particular side.

In past research with VisNet, this single-cell information analysis was used when only one object was presented to the network at a time. Therefore, the maximum information that an ideally developed cell could carry was *log*_2_(number of stimuli). However, in this study, the complete object shape (composed of *n* contours) is presented. Therefore, this is conceptually equivalent to always presenting *n* stimuli simultaneously, thus altering the maximum attainable value of the single-cell information to *log*_2_(*p*) bits of information.

## 3. Results

### 3.1. Study 1: VisNet simulations with artificial visual objects constructed from multiple boundary elements

In Study 1, VisNet was trained on artificial visual objects similar to those shown in Figure 3A. For each simulation, these visual objects had a fixed number of sides (*n*), and the curvature of each side was selected from a fixed number of different boundary conformations or elements (*p*) and were projected on 256 × 256 pixels of simulated retina. Therefore, for each simulation there were *p*^*n*^ complete objects constructed from all combinations of the *n* × *p* contour elements. These artificially constructed objects allowed us to investigate how the learned neuronal response properties are affected by *n* and *p*. We then investigated the development of translation invariance as objects are shifted by 10 pixels at a time over a grid of four different locations on the retina by utilizing the trace learning mechanism discussed above.

#### 3.1.1. Development of neurons that respond to localized boundary conformation

We began by demonstrating how neurons in the output layer learn to respond to individual boundary contour elements when VisNet, implemented with competitive network, is trained on whole objects comprised of a number of such boundary elements. During training, the feed-forward synaptic connections were modified using the Hebb learning rule.

VisNet was first trained on a set of stimuli with *n* = 3 sides: top, left, and right. Each side has two possible boundary conformations: concave and convex. This gave a total of 2^3^ = 8 objects. As conceptually the third layer of VisNet may represent TEO, the VisNet architecture we used consisted of three competitive network layers in this simulation.

**Figure 4:**
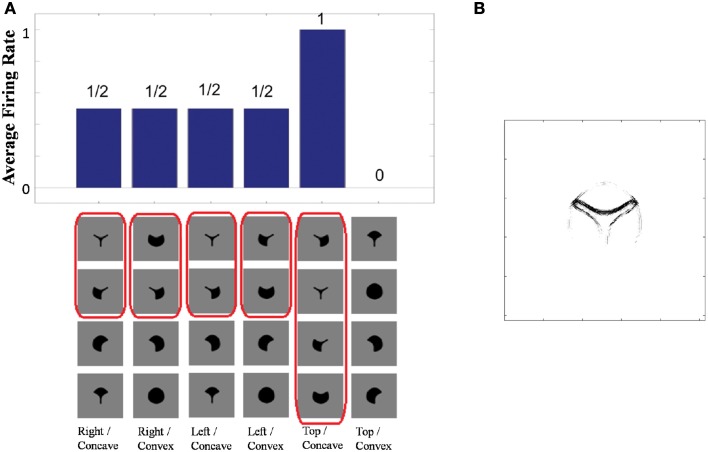
**(A)** The responses of a cell in the output (third) layer of VisNet that developed selectivity to the concave contour on the top of each object. Top: a histogram of the average firing rate responses of the neuron to six (overlapping) subsets of objects. Bottom: the actual subsets of objects that correspond to the six data points shown in the histogram. The objects that the cell responds to are ringed in red. **(B)** The input Gabor filters that an output cell in layer 3 has learned to respond to after training.

Figure 4A shows the learned responses *y*, given by Equation (eqn_firing_rate), of a typical output cell in layer 3 of VisNet, which developed selectivity to a concave contour situated at the top of each object after training; the criteria of the selectivity is whether the cell responds with a firing rate, *r*, approximately equal to 1 (1.00000 ≥ *y* ≥ 0.99995) across a set of whole objects containing a concave contour on the top while the cell responds with a firing rate approximately equal to 0 (0.00005 > *y* ≥ 0.00000) across a set of whole objects not containing a concave contour on the top.

Figure 4A (top) shows a histogram of the average firing rate responses of the neuron to six (overlapping) subsets of objects, where each subset contains all those objects that incorporate a particular one of the six contour elements. Figure 4A (bottom) shows the actual subsets of objects that correspond to the six data points shown in the histogram. The results confirm that the neuron responds selectively.

Figure 4B shows the input Gabor filters that the same output cell in layer 3 has learned to respond to after training. In this case, the neuron receives the strongest inputs from a subset of Gabor filters that represent a concave contour on the top of each object. Such neuronal representations about each contour shape were found across the layer in the trained network. The distribution was quantified later in Sections 3.1.3 and 3.1.4.

#### 3.1.2. How the responses of neurons to their preferred boundary elements depend on the position of the boundary element in the frame of reference of the object

Additional simulations investigated how the responses of neurons to their preferred boundary element depended on the position of the boundary element with respect to the object. In these simulations, VisNet, implemented with competitive networks, was trained on objects constructed with *n* = 4 sides: top, bottom, left, and right. Each side had *p* = 3 possible boundary conformations: concave, straight and convex. During training, the feed-forward synaptic connections were modified using the Hebb learning rule.

**Figure 5:**
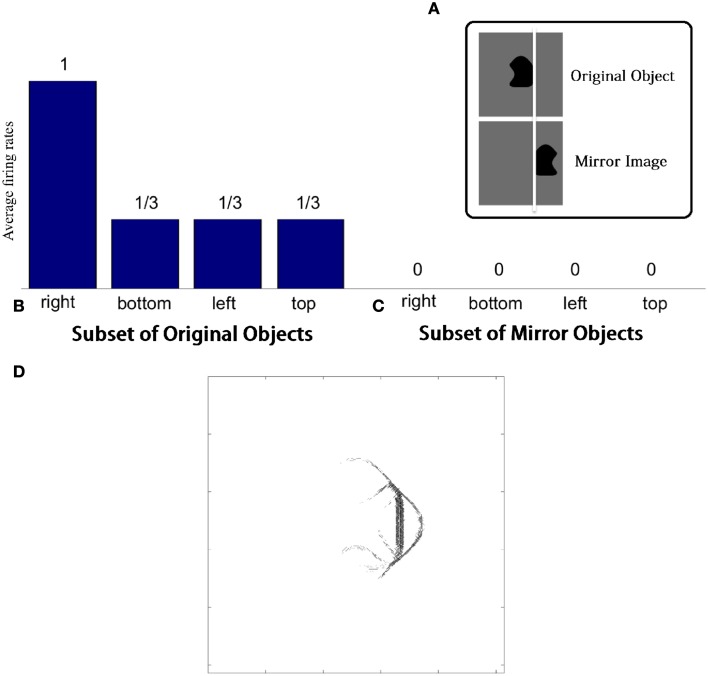
**Demonstration of neuronal response tuning in an object centered frame of reference**. Example stimuli used for testing are shown in **(A)**. **(B)** Shows a histogram of the average firing rate response of the neuron to the four subsets of trained objects that contain a straight contour at one of the sides: right, bottom, left, and top. **(C)** Shows similar results for the mirror image objects. **(D)** The input Gabor filters that had strong connectivity through the layers to a neuron that had learned to respond to a straight contour on the right of each object.

VisNet was tested with two sets of objects. The first set contained those four-sided objects from the original training set that had at least one straight contour element, either on the right, bottom, left, or top. The second set contained mirror images of the first set of objects. The mirror images were constructed by reflecting the original trained objects around the retinal location of the vertical straight contour on the right of the training objects so that the vertical straight contours on the right and left of the two objects are aligned on the retina as shown in Figure 5A. If the neuron has learned about the local image context represented by nearby input filers, the neuron should respond only to the original images with a vertical straight contour on the right.

This effect is confirmed in Figures 5B,C. Figure 5B shows a histogram of the average firing rate response of the neuron to the four subsets of trained objects that contain a straight contour at one of the sides: right, bottom, left, and top (conventions as in Figure 4A). The histogram confirms that the neuron has learned to respond to a vertical straight contour on the right of each of the trained objects. Figure 5C shows similar results for the mirror image objects. Here it can be seen that the neuron fails to respond to any of the mirror image objects, including those mirror image objects with a vertical straight contour on the left.

Figure 5D shows the input Gabor filters that had strong connectivity through the layers to such a neuron. The plot is dominated by a strong vertical straight bar on the right hand side. This shows that the neuron has learned to respond to a straight contour on the right of each object. However, the activity of the neuron will also be influenced by other less strong filters shown in the plot. These additional filters extend furthest to the left of the dominating vertical straight bar. In particular, the strong input filters to the left of the vertical straight bar represent boundary contour features that could co-occur within an object with the vertical straight contour on the right. The same is not true for the curve on the right of the vertical straight bar, which joins the same two vertices linked by the vertical straight bar and so would have to be an alternative contour element to the vertical straight bar. The effect of this pattern of additional input filters is that the neuron may require the presence of additional object contours to the left of the vertical straight contour in order for the neuron to respond. That is, the neuron will only respond to a vertical straight contour when that particular contour shape is on the right hand side of an object rather than the left of the object.

#### 3.1.3. How the number of object sides (*n*) and the number of possible boundary elements at each side (*p*) affect the learned neuronal response properties

We investigated how the neuronal firing properties that develop in the network depend on the number of object sides (*n*) and the number of possible boundary contour elements (*p*) at each side. Each simulation was run with a fixed value of *n* and *p*. Across simulations, the number of sides, *n*, was varied from 3 to 8, while the number of possible boundary elements, *p*, was varied from 2 to 4. For each simulation, the network was trained on the full set of objects that could be constructed given the fixed values of *n* and *p* for that simulation; however, simulations with *p*^*n*^ > 1000 were omitted for practical reasons. During training, the feed-forward synaptic connections were modified using the Hebb learning rule within VisNet implemented with competitive networks.

**Figure 6:**
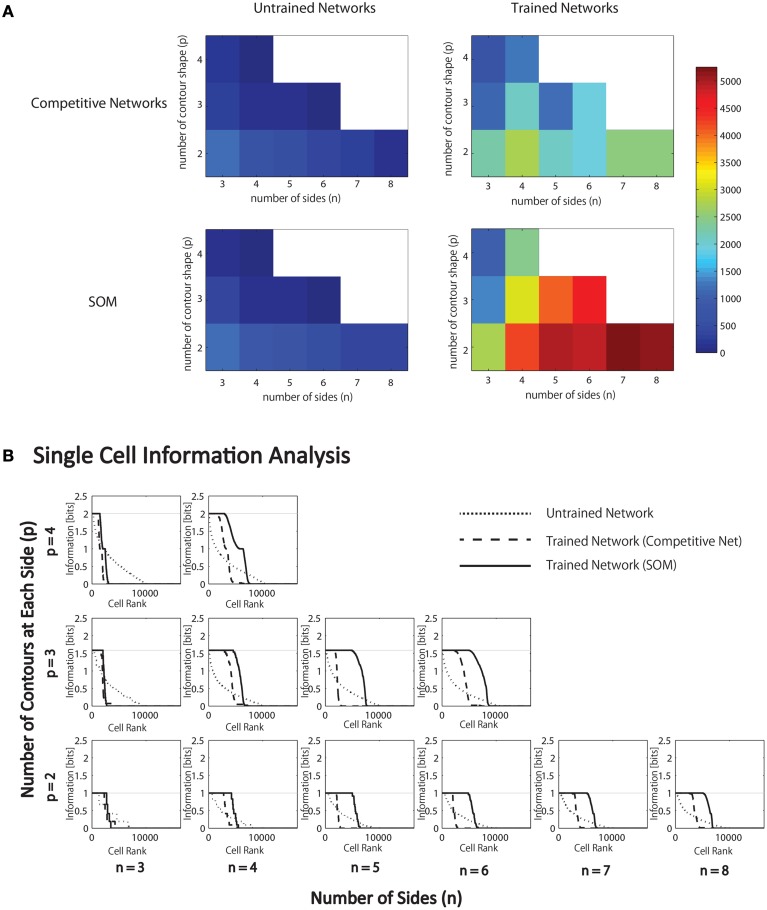
**Results of simulations in which VisNet is trained and tested on objects constructed with a fixed number of sides (*n*) and number of possible boundary elements at each side, *p***. **(A)** For each simulation, the table records the number of neurons (in the third layer) that learned to respond selectively to all objects that contained one particular type of boundary contour element, but not to objects that did not contain that boundary element. Results are given before training (left) and after training (right). **(B)** Single cell information analysis results. For each simulation, the single cell information measures for all output (third) layer neurons are plotted in rank order according to how much information they carry. For each simulation, results are presented before training (dotted line) and after training with competitive network (broken dashed line) and with SOM (solid line).

For each combination of *n* and *p*, Figure 6A (top) gives the number of neurons that learned to respond selectively to all objects that contained one particular type of boundary contour element, but not to objects that did not contain that boundary element.

It was found that the last layer of the untrained network already contained a small number of cells that were selective for objects that contained one type of boundary element. This was because this simulation task was relatively easy in that it did not require the output neurons to respond invariantly as objects were translated across different retinal locations. In simulations reported later in Section 3.1.6, the output neurons were tested with the objects presented in different retinal locations. In these simulations, training was indeed required to produce any neurons that responded selectively to objects containing one kind of boundary element.

In the trained network, it can be seen that all simulations produced large numbers of neurons that were selective for objects that contained one particular type of boundary element. Secondly, the number of object sides, *n*, did not have a significant systematic effect on the performance of the network. In contrast, as the number of possible boundary elements at each side, *p*, increased, the number of neurons that learned to respond selectively to objects containing one type of boundary element declined.

We hypothesize that this is due to the effective increase in the density of the boundary contour elements at each side, which increases the difficulty of neurons in the higher layers developing separate representations of these more similar boundary conformations. In particular, an invariance learning mechanism known as Continuous Transformation (CT) learning (Stringer et al., 2006) may cause neurons in higher layers to learn to respond to a number of similar boundary conformations at each side; CT learning is able to bind smoothly varying input patterns, such as a continuum of different possible boundary conformations at one of the object sides, onto the same postsynaptic neuron. In this way, CT learning may dramatically reduce the selectivity of neurons for particular boundary conformations.

**Figure 7:**
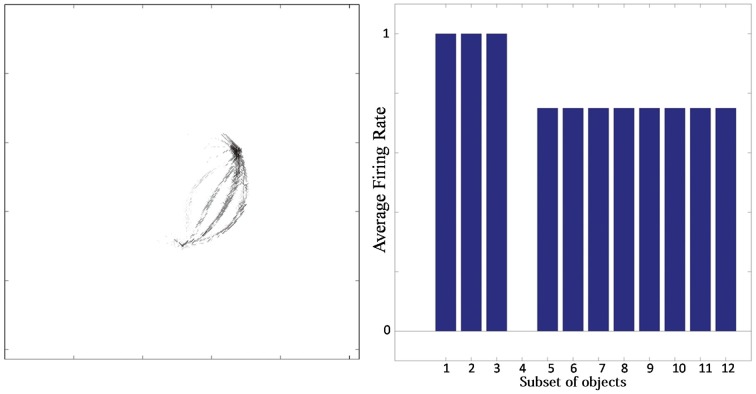
**Simulation showing the failure of an output neuron to discriminate between a relatively large number of boundary contours at one object side. Left:** The input Gabor filters that had strong connectivity through the layers to the output neuron. **Right:** histogram showing average firing rate response of the neuron to the 12 subsets of objects that contain one of the different boundary elements. That is, each of the data points (1–12) represents the average firing rate of the neuron across the 16 objects containing the following boundary elements: (1) right/sharp-convex, (2) right/convex, (3) right/concave, (4) right/sharp-concave, (5) left/sharp-convex, (6) left/convex, (7) left/concave, (8) left/sharp-concave, (9) top/sharp-convex, (10) top/convex, (11) top/concave, (12) top/sharp-concave.

Typical network behavior for a relatively large value of *p* is shown in Figure 7. In this example, the network was trained on objects with *n* = 3 sides, each of which had *p* = 4 possible boundary elements. The figure shows results for a typical output cell that failed to learn to respond selectively to objects containing one particular type of boundary contour. Figure 7 (left) shows the input Gabor filters that had strong connectivity through the layers to the neuron. The neuron has strong connections from three similar boundary elements on the lower right. Figure 7 (right) shows the average firing rate response of the neuron to the 12 subsets of objects that contain one of the different boundary elements. The neuron responds maximally to the first three subsets of objects, which contain the three boundary elements that are strongly represented in the left plot. Thus, the neuron has learned to respond equally strongly to all of these three boundary elements and is unable to distinguish between them. This observed behavior is typical when the number (density) of boundary contour elements at each side is increased. Investigation into the responses of neurons across the output layer after the training the network on objects where each side had a relatively high number of possible boundary element contours, *p*, showed that many cells were unable to distinguish between differently shaped contours on the same sides.

The simulations at this juncture show that a biologically plausible neural network can learn to code relative position information for visual elements, but has limited capacity. In the next section, we show how introducing a SOM architecture within each layer of VisNet can enhance the selectivity of neurons for individual boundary elements when the number of boundary elements at each side, *p*, is large, overcoming the capacity limitation.

#### 3.1.4. The effect of a Self-Organizing Map (SOM) architecture on learned neural selectivity for boundary contour elements

We compared the performance of the standard competitive network architecture in each layer with performance when a SOM was introduced. We hypothesized that the SOM architecture could increase the capacity of the network to represent and distinguish between a larger number of finer variations in boundary contour curvature.

As discussed in the previous section, a competitive network may have difficulty in forming separate output representations of similar input patterns. In particular, CT learning (Stringer et al., 2006) may encourage the same output neurons to learn to respond to similar input patterns representing boundary contour elements of slightly different shape, or even bind together a continuum of input patterns covering the space of all possible boundary shapes at a particular object-centered boundary location.

The SOM architecture is specifically designed to encourage the output neurons to develop a fine-scaled representation of a continuum of smoothly varying input patterns (Kohonen, 2000). A SOM has additional short range lateral excitatory connections between neurons within each layer. These connections encourage nearby output neurons to learn to respond to similar input patterns, which in turn leads to a map-like arrangement of neuronal response characteristics across the layer after training. In particular, slightly different input patterns will be distributed across different output neurons. Thus, the effect of these additional short range excitatory connections is to influence learning in the network to spread the representations of a continuum of overlapping input patterns over a map of output neurons. This should allow the network to develop a more fine-grained representation of the space of possible boundary contour shapes.

We therefore hypothesized that the introduction of a SOM architecture within each layer of VisNet would spread out the representations of many different boundary contour curvatures (*p*) at a particular side of the object over a map of output neurons. This would help to produce distinct neural representations of a large number of different boundary contour elements in the output layer, and effectively increase the capacity of the network to represent finer variations in boundary contour curvature.

During training, the feed-forward synaptic connections were, again modified using the Hebb learning rule, and the simulation results with the SOM architecture implemented within each layer are presented in Figure 6A (bottom). The network was tested on objects constructed with a fixed number of sides, *n*, and different numbers of possible boundary elements at each side, *p*. For each simulation, the heatmap shows the number of neurons that learned to respond selectively to all objects that contained one particular type of boundary contour element, but not to other objects. These results should be compared with Figure 6A (top), which gives the corresponding results with a competitive network architecture implemented within each layer. As hypothesized, the introduction of SOM architecture within each layer led to many more neurons learning to respond selectively to objects containing a particular boundary contour element. This effect is particularly pronounced for larger numbers of *n* and *p*.

These effects can also be seen by examining the amount of information carried by neurons about the presence of particular types of boundary elements within the objects presented to VisNet. We have previously used information theoretic measures to assess the amount of information carried by neurons about the presence of whole object stimuli within a scene, where the objects may be presented under different transforms such as changes in retinal position or orientation (Wallis and Rolls, 1997; Rolls and Milward, 2000; Stringer et al., 2007; Stringer and Rolls, 2008). A neuron that responds selectively to one particular stimulus across a large number of transforms will carry a high level of information about the presence of that object within a scene. In this current paper, we were instead interested in the amount of information carried by neurons about the presence of particular boundary elements within an object.

Figure 6B present the single cell information analysis results for simulations in which VisNet was tested on objects with different numbers of sides, *n*, and numbers of possible boundary elements at each side, *p*. The results are presented before training (dotted line), after training with the competitive network architecture (broken dashed line) and with the SOM architecture (solid line). The single cell information measures for all output layer neurons are plotted in rank order according to how much information they carry. In all simulations, training the network on the set of *p*^*n*^ whole objects led to many top layer neurons attaining the maximal level of single cell information of *log*_2_(*p*) bits. These results imply that training the network on the whole objects led to many output neurons learning to respond selectively to all of the objects that contained a particular one of the boundary contour elements, but not to objects that do not contain that boundary element. That is, these neurons had learned to respond to the presence of that particular boundary contour element within any object. In all simulations, many top layer neurons attained the maximal level of single cell information of *log*_2_(*p*) bits. However, consistent with our hypothesis, the incorporation of a SOM architecture typically led to a significant increase in the number of neurons that attained the maximal level of single cell information.

**Figure 8:**
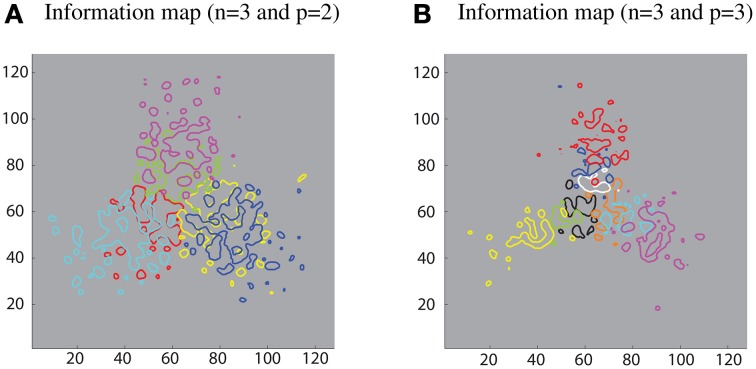
**Simulation results demonstrating that the SOM architecture leads to a feature map in the output layer**. **(A)** Left: contour plots showing the amount of single cell information carried by the 128 × 128 layer of output neurons for six boundary elements after training on objects with *n* = 3 and *p* = 2. The different colored contour plots correspond to the following boundary elements: top/convex (pink), top/concave (light green), right/convex (blue), right/concave (yellow), left/convex (light blue), and left/concave (red). **(B)** Right: similar results for the case *n* = 3 and *p* = 3.

Furthermore, different sub-populations of cells that carry maximum single-cell information about each contour element were mapped onto the corresponding locations within the layer. This extended analysis has revealed that using a SOM led to a feature map as shown in Figure 8. This result was consistent with various physiological findings that indicate the topographic organization within ventral visual pathway (Larsson and Heeger, 2006; Hansen et al., 2007; Silver and Kastner, 2009).

#### 3.1.5. Response properties of neurons through successive layers of VisNet

We subsequently investigated how the response properties of neurons vary through successive layers of VisNet, which is implemented with SOM, before and after training. For all of the simulations performed, the feed-forward synaptic connections were modified using the Hebb learning rule.

Table 6 presents simulation results showing the responses of neurons through layers 1 to 3. The results are presented for a simulation with *n* = 4 sides and *p* = 2 contour elements per side and compared before and after training. Each sub-table gives the number of neurons that responded selectively to either objects containing a single boundary element, objects containing a combination of two boundary elements, or a single whole object. It can be seen that, in all three layers, training the network led to a substantial increase in the number of neurons that responded to objects containing a single boundary element. The numbers of neurons that learned to respond to individual boundary elements increased through successive layers of VisNet.

**Table 6:** **Simulation results showing the responses of neurons through layers 1 to 3 with the SOM architecture**.

	**N4P2 experiment (SOM)**			
**Layer**	**1 Contour**	**2 Contours**	**Object**
**UNTRAINED NETWORK**			
3	856	270	538
2	418	104	82
1	293	0	0
**TRAINED NETWORK**			
3	4216	92	89
2	2440	10	10
1	540	8	0

**Figure 9:**
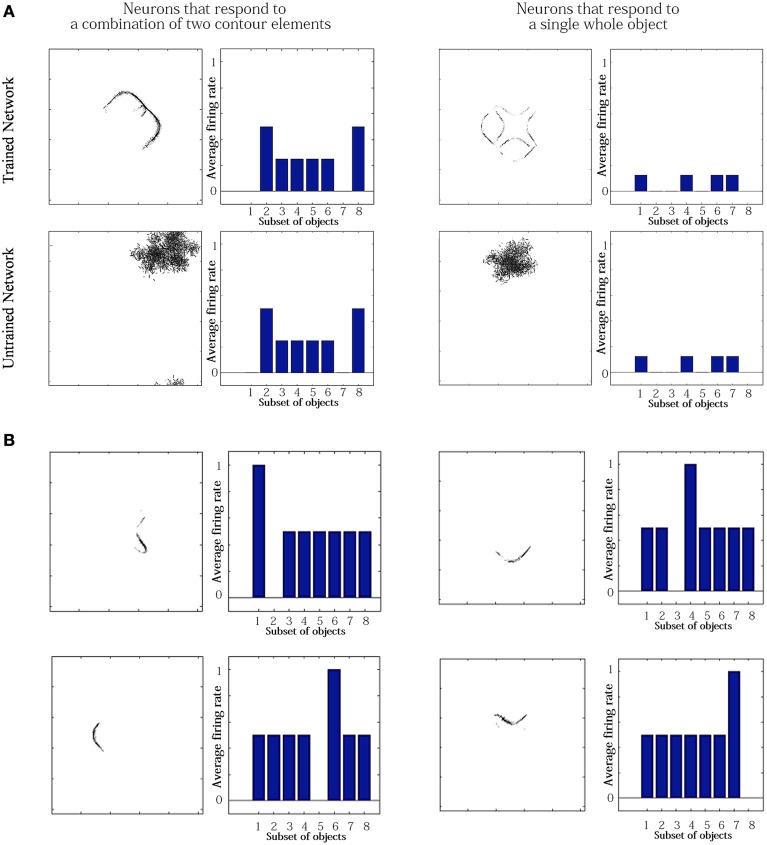
**Comparison of response properties of trained and untrained neurons in simulations with the Self-Organizing Map (SOM) architecture**. The network is presented with objects containing *n* = 4 sides, where each side has *p* = 2 possible boundary elements. **(A)** The figure shows results for four typical neurons. For each neuron, we show two plots. Left: The input Gabor filters that had strong connectivity through the layers to the neuron. Right: histogram showing average firing rate response of the neuron to the eight subsets of objects that contain one of the different boundary elements. That is, each of the data points (1–8) represents the average firing rate of the neuron across the eight objects containing the following boundary elements: (1) right/concave, (2) right/convex, (3) bottom/concave, (4) bottom/convex, (5) left/concave, (6) left/convex, (7) top/concave, (8) top/convex. **(B)** Four neurons in layer 2 with strong synaptic connections to the output neuron shown in top-right in **(A)**.

For the simulation reported in Table 6, training did not lead to a similarly large increase in the numbers of neurons that responded to either a combination of two boundary elements, or a single whole object. This contrasts with experimental studies showing that neurons in the later stages of the ventral visual pathway, TEO and posterior TE, integrate information from multiple boundary contour elements (Brincat and Connor, 2004). We, therefore, investigated how neurons might learn to respond to localized clusters of boundary contour elements and also to whole objects. In fact, by examining the input Gabor filters that had a strong connectivity to these types of neuron, we were able to show that some neurons in VisNet were indeed learning to respond to either a combination of two boundary elements, or a whole object. These results are shown in Figure 9A.

Figure 9A compares the response properties of trained and untrained neurons in simulations with the SOM architecture. The network is presented with objects containing *n* = 4 sides, where each side has *p* = 2 possible boundary elements. Results are shown for four neurons. For each neuron, we show the input Gabor filters that had strong connectivity through the layers to the neuron (left), and a histogram showing average firing rate response of the neuron to the objects that contain one of the 8 boundary elements (right). The four neurons shown in the Figure 9A had the following characteristics. (top-left) A trained neuron that has learned to respond to a combination of two adjacent boundary contour elements: top convex and right convex. The Gabor filter plot shows that the feed-forward synaptic weights have been strengthened selectively from the two boundary elements only. (top-right) A trained neuron that has learned to respond to a whole object. The preferred object is comprised of two concave on top and right and two convex on bottom and left. The Gabor filter plot shows that the neuron has learned to respond to the complete set of boundary elements comprising the preferred object. (bottom-left) An untrained neuron that happens to respond selectively during testing to two adjacent boundary elements. However, the Gabor filter plot shows that a random collection of Gabor filters have strong feed-forward connections to the neuron. This means that across a richer diversity of test images, this neuron would not maintain such a strict selectivity, and would in fact be most effectively stimulated by the random constellation of Gabor filters shown. (bottom-right) An untrained neuron that responds selectively to a whole object. The Gabor filter plot shows that the neuron receives strong connections from a random collection of Gabor filters. This neuron would not maintain a strict selectivity to the object when tested on a greater diversity of images.

The conclusion of the results shown in Figure 9A is that although Table 6 appeared not to show an increase during training in the numbers of neurons that responded to combinations of two boundary elements or a whole object, in fact training did lead to an increase in the numbers of neurons that had specifically learned to respond to whole stimuli. However, in Table 6, this effect had been masked by the existence of many untrained cells that already responded by chance to combinations of two boundary elements or a whole object, but which in fact had random inputs from a large randomized collection of Gabor filters. Such untrained neurons are unlikely to be selective for combinations of two boundary elements or a particular object if the network were tested on a richer diversity of images. In particular, these untrained neurons would respond more selectively for images corresponding to the random constellations of Gabor filters shown in the bottom of Figure 9A. In contrast, the trained neurons on the top have strengthened connections specifically from combinations of two boundary elements or a whole object, and would therefore maintain their selectivity more robustly across a greater variety of test images.

We also found that output neurons in layer 3 learned to respond to whole objects by combining inputs from neurons in the preceding layer that responded to the individual boundary elements. This can be seen by examining the strengths of the synaptic connections from neurons in layer 2 to output neurons in layer 3 after training. Output neurons that had learned to respond to a particular object received the strongest synaptic connections from neurons in layer 2 that represented the constituent boundary elements of that object. Figure 9B shows four neurons in layer 2 with strong synaptic connections to a whole shape selective neuron reported in the top-left of Figure 9A. the output neuron shown in Figure 9A. Each of the four neurons in layer 2 had learned to respond to a different one of the boundary elements which were contained in the object that the output neuron had learned to respond to. This example shows that neurons in the later stages of the model are able to integrate information from multiple boundary contour elements, as consistent with neurophysiological results for areas TEO and posterior TE of the primate ventral visual pathway (Brincat and Connor, 2004).

#### 3.1.6. Translation invariance of neuronal responses as objects are shifted across different locations on the retina

The neurons reported by Pasupathy and Connor (2001) in area V4, and neurons reported by Brincat and Connor (2004) in areas TEO and posterior TE, respond with translation invariance as an object is shifted across different retinal locations. In this section we show how these translation invariant neuronal responses may be set up by training the network with the trace learning rule. The trace learning rule encourages individual postsynaptic neurons to learn to respond to subsets of input patterns that tend to occur close together in time. Therefore, in the simulation described below, during training we selected each object in turn and presented that object in a number of different retinal locations before moving on to the next object.

For this simulation, VisNet had four layers with a SOM architecture implemented within each layer. The visual objects had *n* = 4 sides, where each side has *p* = 3 possible boundary elements. Each of the visual objects was presented in a 2 × 2 grid of four different retinal locations, which were separated by horizontal and vertical shifts of 10 pixels.

**Figure 10:**
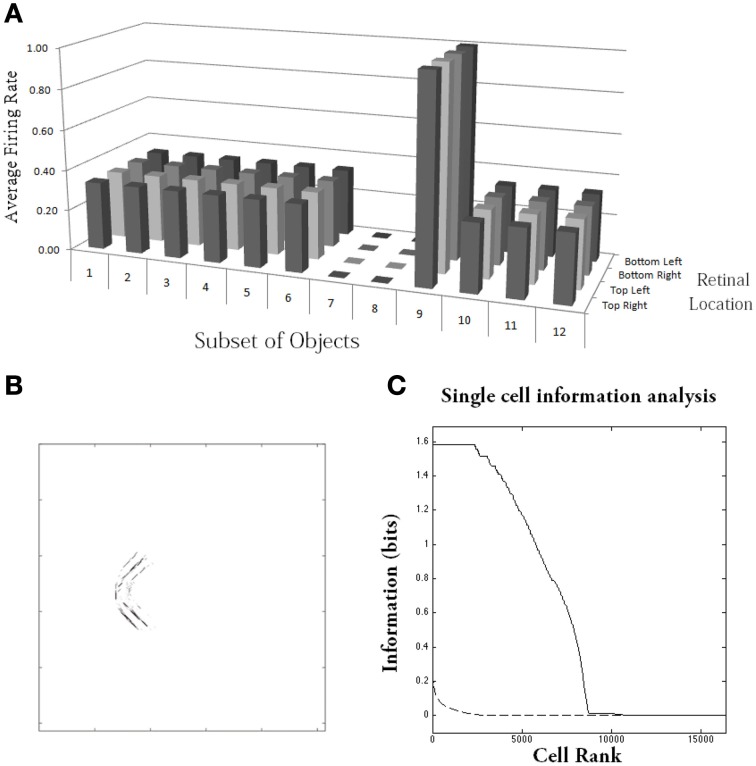
**Simulation of network trained with the trace learning rule as each of the visual objects is shifted across 4 different retinal locations: top right, top left, bottom right and bottom left**. The objects had *n* = 4 sides, where each side has *p* = 3 possible boundary elements. The figure shows results after training for a typical output neuron in layer 4. **(A)** Histogram showing the average firing rate response of the output neuron to the 12 subsets of objects that contain one of the boundary contour elements. That is, each of the data points (1–12) represents the average firing rate of the neuron across the 27 objects containing the following boundary elements: (1) right/concave, (2) right/straight, (3) right/convex, (4) bottom/concave, (5) bottom/straight, (6) bottom/convex, (7) left/concave, (8) left/straight, (9) left/convex, (10) top/concave, (11) top/straight, (12) top/convex. Each of these results is given for the objects placed in the four different retinal locations. **(B)** The input Gabor filters that had strong connectivity through the layers to the output neuron. **(C)** Single cell information analysis of a simulation where visual object, which has *n* = 4 sides, where each side has *p* = 3 possible boundary elements, is shifted across four different retinal locations. The single cell information measures for all output layer neurons are plotted in rank order according to how much information they carry. Results are presented before training (broken line) and after training (solid line).

Figure 10A shows the results after training for a typical output neuron in layer 4. Figure 10B shows the input Gabor filters that had strong connectivity through the layers to the output neuron. It can be seen that the neuron has strong connections from a convex boundary element on the left of an object. The separate contours that can be seen in the plot correspond to the different retinal locations in which the objects are trained. Figure 10A shows a histogram presenting the average firing rate response of the output neuron to the 12 subsets of objects that contain one of the boundary contour elements. The neuron responds maximally to the subset of objects containing a convex boundary element on the left. Notably, the neuron responds maximally to this subset of objects over all four retinal locations. Thus, the neuron has learned to respond to objects containing the convex boundary element on the left regardless of where the object is presented on the retina. These translation invariant neuronal responses are a result of training the network with the trace learning rule.

Figure 10C shows findings from the single cell information analysis. The results are presented before training (broken line) and after training (solid line). Training the network on the set of *p*^*n*^ whole objects over the four retinal locations led to many top layer neurons attaining the maximal level of single cell information of *log*_2_(*p*) bits. Neurons carrying maximal single cell information responded selectively to a subset of objects containing one particular type of boundary element, and with translation invariance as the objects also were shifted over all four retinal locations. In these simulations with translation invariance, the information is dramatically increased after training. This is because it is very unlikely for untrained neurons to both respond selectively to a single boundary contour element across all objects, and be able to respond with translation invariance as these objects are shifted across the retina. Therefore, training will lead to a much more significant difference between the performances of the untrained and trained networks.

### 3.2. Study 2: VisNet simulations with visual stimuli of pasupathy and connor

In Study 2, the visual stimuli presented to VisNet were similar to the artificial stimuli used in the neurophysiological experiments of Pasupathy and Connor (2001) shown in Figure 3B. This allowed direct comparison between the learned response characteristics of the neurons in the VisNet model and the experimentally observed cell responses encoding local boundary information reported.

The stimuli were constructed by systematically combining sharp convex, medium convex, broad convex, medium concave and broad concave boundary elements to form closed shapes. We also vary the angular separations of the vertices used to construct the stimuli on 256 × 256 pixels of the simulated retina as shown in Figure 3B. Furthermore, we also rotated the visual stimuli through 360° in a single central location on the retina in steps of 10° during training to provide more natural visual training. This meant that there was not such a clean statistical decoupling between the boundary elements as for Study 1. Nevertheless, we expected that with the new objects used in Study 2 there would still be sufficient statistical decoupling between the boundary elements to ensure that the network developed neurons during visually guided learning that responded to a localized region of boundary curvature.

For all simulations in Study 2, the VisNet architecture consisted of three layers of SOM, where each layer is composed of 64 × 64 neurons. During training, the feed-forward synaptic weights are modified using the trace learing rule, which is needed to develop translation invariant neuronal responses.

#### 3.2.1. Development of neurons encoding local boundary conformation in an object-centered frame of reference

**Figure 11:**
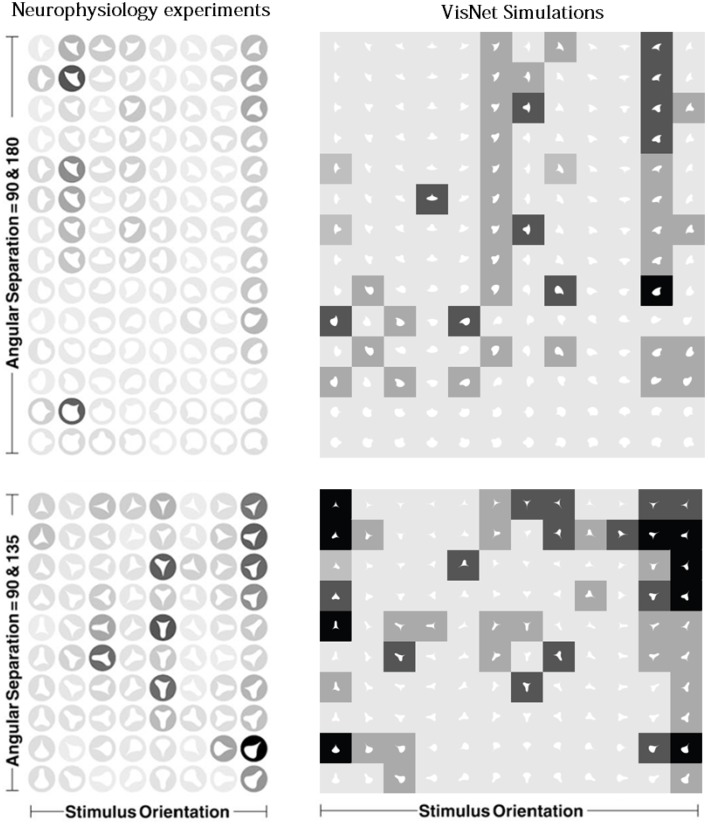
**Comparison between the single neuron recording data of Pasupathy and Connor (2001) and corresponding results from VisNet simulations**. On the left of the figure are shown the responses of a neuron recorded in area V4 of the primate ventral visual pathway and shown in Figure 5A of Pasupathy and Connor (2001). Each object shape shown to the monkey is represented by a white icon, and the firing rate response of the neuron is represented by the surrounding shading with high firing denoted by black. Each row shows a different object shape, with each column corresponding to a different orientation of the object. It can be seen that the neuron responds selectively to object shapes with an acute convex curvature at the top right of the object. On the right of the figure are shown corresponding results for an output cell in layer 3 of VisNet, which has learned to respond with similar selectivity.

Figure 11 shows a comparison between the responses of a neuron recorded in area V4 of the primate ventral visual pathway by Pasupathy and Connor (2001) and a neuron recorded from our simulation, which exhibits a similar degree of selectivity. The neuron recorded by Pasupathy and Connor (2001) responds selectively to object shapes with an acute convex curvature at the top right of the object. Many other neurons in the output layer of VisNet learned to respond selectively to particular combinations of local boundary curvature and position with respect to the center of mass of the object. The network accomplished this even though the statistical independence of the boundary contour elements was not perfect.

**Figure 12:**
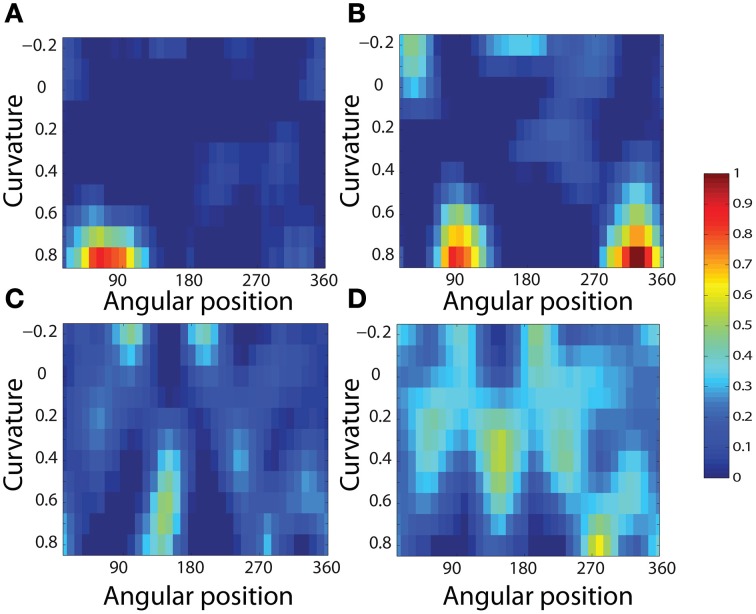
**Heatmap showing the average responses of four output neurons to different combinations of local boundary curvature and angular position where the boundary curvature appears**. The average is computed over all orientations (0°–360°) of all objects. **(A)** The neuron responds maximally to object shapes with an acute convex curvature at the top-right. This is the same neuron that was shown on the right of Figure 11. **(B–D)** Three other cells that show different firing patterns are also plotted to show the variability in the network.

To analyse the detailed firing properties of each output neuron and quantified the distributions, we recorded its response to all objects as they were rotated through 360°. Next we segmented the boundary contour of each object into multiple elements based on the positions where the rate of change of the curvature exceeded a fixed threshold. This then enabled us to calculate the average response of the neuron to each particular combination of local boundary curvature and angular position where that boundary curvature appears, where the average is computed over all orientations of all objects. Figure 12A shows a heatmap of the average responses of the output neuron shown on the right of Figure 11 to different combinations of boundary conformation and angular position. The result indicates that this neuron responds maximally to object shapes with an acute convex curvature at the top right. The correlation coefficient between the result and a predicted result of a modeled V4 neuron based on Gaussian distribution, which is tuned to acute contours at 70° is strong (0.798) and confirms the selectivity. Figures 12B–D show examples of different trained cells.

For each neuron, we then analyzed the number of local peaks in the heatmap of average firing rate against curvature and angular position, as shown in Figure 12. Specifically, for each neuron we counted the number of local peaks that were greater than 60% of the average firing rate across the heatmap. Before training, 176 cells had one peak, 98 cells had two peaks, 63 cells had three peaks, and 44 cells had four peaks. After training, the distributions were 319 cells, 460 cells, 414 cells, and 374 cells. (These distributions were significantly different, χ^2^ = 17.58, *df* = 3, *P* ≪ 0.01.) Thus, training led to a large increase in the number of neurons that were selectively tuned to either one or just a few boundary contour elements. The simulation results also predict the existence of individual neurons that are tuned to boundary elements in multiple locations. Consistent with this, Brincat and Connor (2004) have reported that some neurons in TEO and posterior TE do indeed respond to the co-occurrence of multiple adjacent contour elements.

#### 3.2.2. Development of translation invariant neuronal responses

Pasupathy and Connor (2001) and Brincat and Connor (2004) reported that neurons encoding the boundary conformation of objects also respond with translation invariance as an object is shifted across different retinal locations. In this section we confirm that neurons in VisNet also develop translation invariant responses when the network is trained on the stimuli shown in Figure 3B. To cope with the larger computational resource requirements, only the stimuli with an angular separation between vertices of 135°∕135°∕90° were used, and the size of the image was reduced to 128 × 128 pixels. During training, the trace learning rule was used to modify the synaptic weights.

In this simulation, during training each object was shifted across a 3 × 3 grid of nine different retinal locations, which are separated by horizontal and vertical intervals of 10 pixels. At each pixel location, the objects are presented in all orientations through 0°–360° in 10° steps. This means that during training the objects underwent two different kinds of transformation, both translation and rotation. We assume that typically the eyes shift about a visual scene more rapidly than the objects rotate on the retina. To simulate this effect, VisNet was trained as follows. During training, the orientation of each object was kept fixed at some initial angle while the object was shifted across all of the different retinal locations. Then the orientation of the object was adjusted by, for example, 10° and the object was again shifted across all of the retinal locations. This procedure was repeated for all object orientations from 0° to 360° in steps of 10°. This training procedure ensured that images of each object in the same orientation but different retinal locations were closely clustered together in time.

**Figure 13:**
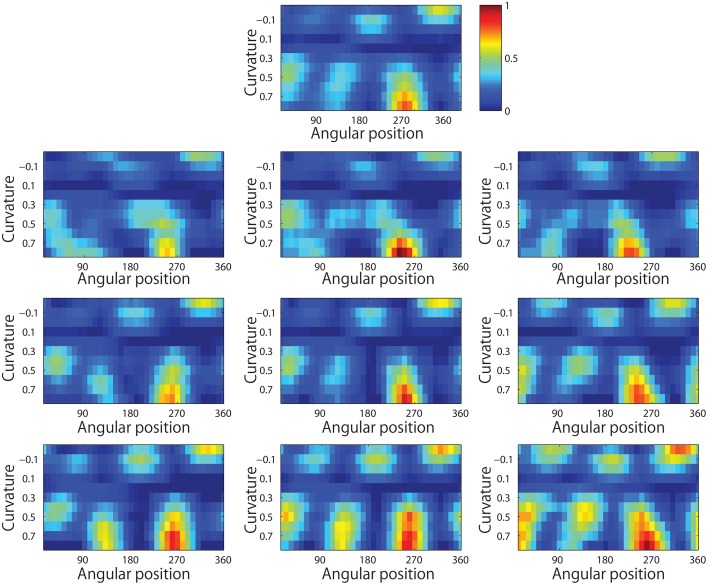
**Demonstration of translation invariance after training the network on the visual stimuli shown in Figure 3B**. The figure shows the average responses of a typical output neuron over the nine retinal locations after training with the trace learning rule. Each of the ten subplots shows the average responses of the neuron to different combinations of local boundary curvature and angular position where the boundary curvature appears. The top subplot shows the average neuronal responses computed over all nine retinal locations. While the bottom subplots show the average neuronal responses computed separately for each of the nine retinal locations.

Figure 13 shows results for a typical output neuron after training. Each subplot shows the average responses of the neuron to different combinations of local boundary curvature and angular position. The top subplot shows the average neuronal responses over all nine retinal locations, while the remaining subplots show the average neuronal responses to each of the nine separate retinal locations.

In order to quantify the distribution of such cells, the number of peaks of responses for each cell were calculated. Before training, 91 cells had one peak, 61 cells had two peaks, 34 cells had three peaks, and 24 cells had four peaks. After training, the distributions were 288 cells, 253 cells, 119 cells, and 158 cells. (These distributions were significantly different, χ^2^ = 1.99e+03, *df* = 3, *P* ≪ 0.01.)

It is evident that the neuron displays a pattern of selectivity for boundary curvature and angular position that is similar across the nine retinal locations. Thus, the responses of the neuron exhibit translational invariance, similar to the neurons reported in the neurophysiology experiments of Pasupathy and Connor (2001) and Brincat and Connor (2004).

### 3.3. Study 3: VisNet simulations with images of natural objects

In Study 3, VisNet was trained with images of natural objects in order to demonstrate that the learning mechanisms elucidated in this paper and tested with artificially constructed visual stimuli in sections of Study 1 and 2 will indeed work effectively on real world visual objects. We hypothesize that across many images of natural objects with different boundary shapes, there will be an effective statistical decoupling between localized boundary elements, which are defined by local curvature and angular position with respect to the center of mass of the object. This should force the neurons in higher layers of the network to learn to respond to the individual boundary elements rather than the whole objects.

Some examples of the natural objects used in these simulations are shown in Figure 3C. The set of stimuli used in the simulations is composed of 177 realistic three dimensional objects. Various kinds of three dimensional objects are downloaded from Google 3D Warehouse, converted into gray-scaled images, and rescaled to fit on the center of 256 × 256 retina. In order to enhance the realism of the visual images used to train VisNet, during training each of the natural objects is rotated in plane through 360° in steps of 10°. After training, the neuronal responses in the network were examined with the test stimuli used for Study 2 (Figure 3B).

#### 3.3.1. Development of neurons encoding local boundary conformation in an object-centered frame of reference

**Figure 14:**
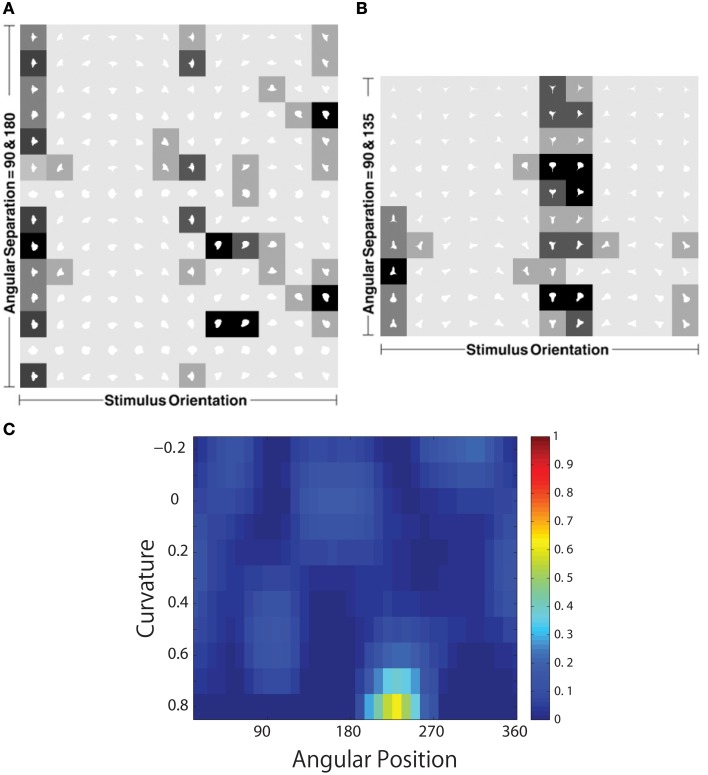
**The development of neuronal responses in VisNet that encode combinations of local boundary curvature and rotational position after the network has been trained with images of natural objects as shown in Figure 3C**. The figure shows the responses of an output neuron which has learned to respond to an acute convex curvature at the bottom left (225°) of an object. **(A,B)** Shows the responses of the neuron to objects with an angular separation between the vertices of 135°∕135°∕90° and 180°∕90°∕90°, respectively. Each object shape is represented by a white icon, and the firing rate response of the neuron is represented by the surrounding shading with high firing denoted by black. Each row shows a different object shape, with each column corresponding to a different orientation of the object. **(C)** Shows a heatmap of the average responses of the neuron to different combinations of local boundary curvature and angular position where the boundary curvature appears. The average is computed over all orientations (0°–360°) of all objects.

Figure 14 shows the responses of a typical output neuron after training. This neuron learned to respond to an acute convex curvature at the bottom left of an object. Moreover, although not shown, many other neurons in the output layer of VisNet learned to respond selectively to particular combinations of local boundary curvature and angular position of the boundary element.

In order to quantify the distribution of such cells, the number of peaks of responses for each cell were calculated. Before training, 176 cells had one peak, 98 cells had two peaks, 63 cells had three peaks, and 44 cells had four peaks. After training, the distributions were 232 cells, 141 cells, 125 cells, and 103 cells. (These distributions were significantly different, χ^2^ = 176.82, *df* = 3, *P* ≪ 0.01.)

This result showed that VisNet was able to develop these neuronal responses even though the network had been trained on many natural visual objects without artificially constructing the boundary shapes from artificially predefined elements.

#### 3.3.2. Development of translation invariant neuronal responses

We then tested whether neurons in VisNet can also develop translation invariant responses when the network was trained on the natural objects shown in Figure 3C. Each of the natural objects was shifted across a 3×3 grid of nine different retinal locations, which were separated by horizontal and vertical intervals of 10 pixels. At each pixel location, the objects were presented in different orientations through 0°–360° in 10° steps. The temporal sequencing of these two kinds of transforms was the same as described in Section 3.2.2. During training, the trace learning rule was used to modify the synaptic weights.

**Figure 15:**
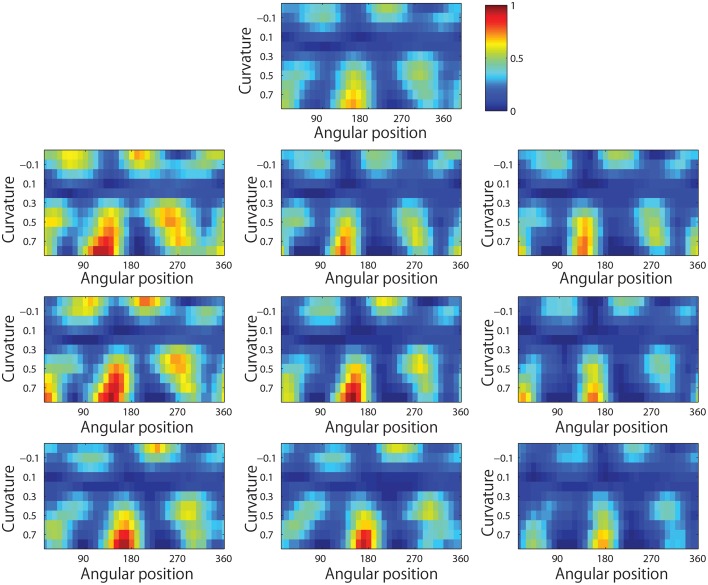
**The development of translation invariant neuronal responses in the output layer of VisNet after the network has been trained with images of natural objects as shown in Figure 3C**. The network was trained with the trace learning rule in order to promote invariance learning across nine different retinal locations. The figure shows the average responses of a typical output neuron over the nine retinal locations after training. Each of the ten subplots shows the average responses of the neuron to different combinations of local boundary curvature and angular position where the boundary curvature appears. The top subplot shows the average neuronal responses computed over all nine retinal locations. While the bottom subplots show the average neuronal responses computed separately for each of the nine retinal locations.

Figure 15 shows results for a typical output neuron after training. Each subplot shows the average responses of the neuron to different combinations of local boundary curvature and angular position. The top subplot shows the average neuronal responses over all nine retinal locations, while the remaining subplots show the average neuronal responses to each of the nine separate retinal locations. It can be seen that the neuron responds selectively to objects with a high convex curvature at the top-left. Moreover, the responses of the neuron are similar across all nine retinal locations.

In order to quantify the distribution of such cells, the number of peaks of responses for each cell were calculated. The distributions were that before training, 97 cells had one peak, 38 cells had two peaks, 25 cells had three peaks, and 31 cells had four peaks, whereas after training, the distributions were 349 cells, 148 cells, 90 cells, and 109 cells. (These distributions were significantly different, χ^2^ = 1.34e + 03, *df* = 3, *P* ≪ 0.01). Thus, the responses of the neuron are reasonably translation invariant, similar to the neurons reported in the neurophysiology experiments of Pasupathy and Connor (2001) and Brincat and Connor (2004).

In conclusion, the above results thus demonstrate that even when VisNet is trained on realistic natural visual objects, where the boundary shapes have not been carefully constructed from a pool of artificial elements, the network still develops neurons that respond selectively to the curvature and location of localized boundary contour elements in the frame of reference of the object. Moreover, with the help of the trace learning rule, these neuronal responses are also translation invariant as an object shifts across different retinal locations.

## 4. Discussion

In this paper, we have demonstrated that when a neural network model, VisNet, of the primate ventral visual pathway is trained on many objects with different boundary shapes, the neurons in the higher layers of the network learn to respond to localized boundary contour elements, which are defined by the curvature and location of the boundary element in the frame of reference of the object. Interestingly, neurons learn to respond to these boundary elements rather than learning to respond to the whole objects that were actually presented during training. Moreover, the neurons were able to learn to respond with translation invariance as visual objects are shifted across different retinal locations. This was shown to be successful when VisNet was trained with either the artificially constructed visual stimuli used in Studies 1 and 2, or with images of natural visual objects in Study 3.

The primary contribution of this paper is to elucidate and test two key biologically plausible learning mechanisms that can combine to promote the development of these neuronal response characteristics. First, similar to the results shown in the previous study with multiple-objects (Stringer et al., 2007; Stringer and Rolls, 2008), if the network is trained on many objects with different boundary shapes, where each boundary is comprised of a different constellation of contour elements, then this leads to a statistical decoupling between the boundary elements. This is sufficient to allow the competitive layers of VisNet to develop neurons that respond to individual boundary elements defined by curvature and position within the object, which are similar to the neurons reported in the physiological experiments conducted by Pasupathy and Connor (2001). Secondly, consistent with previous simulation studies (Wallis and Rolls, 1997; Rolls and Milward, 2000), neurons learned to respond with translation invariance across different retinal locations through the use of a trace learning rule. This kind of learning places constraints on the statistics of how the eyes move and visual objects change or transform on the retina. These two mechanisms together provide a biologically plausible account of how neurons in the primate ventral visual pathway may learn to represent localized boundary contour elements of objects as revealed by Pasupathy and Connor (2001).

Furthermore, neurophysiological experiments carried out by Brincat and Connor (2004) have shown that neurons in the later stages of the ventral visual pathway, TEO and posterior TE, integrate information from multiple boundary contour elements. In our simulations, the number of cells that were tuned to combinations of multiple contours increased in the higher layers. Tracing back the feed-forward synaptic connectivity to these output neurons confirmed that their selectivities were built by combining inputs from neurons representing each local boundary contour in the preceding layer.

The simulations reported in this present work are the first to show how neuronal responses encoding the local boundary conformation of objects may develop through a biologically plausible process of visually-guided learning. Both the Hebb learning rule and trace learning rule used above are biologically plausible in that they are “local” learning rules, which only use locally available biological quantities, such as the activity of the pre- and post-synaptic neurons, to modify the synaptic weights. This is in sharp contrast to other modeling studies that manually set up the synaptic weights in a non-local manner. In particular, the trace learning rule drives the development of translation invariant neuronal responses. Convincing experimental evidence for the presence of trace learning in the primate visual system has been provided by Cox et al. (2005), and a plausible account of the synaptic basis of trace learning has been provided by simulations of biologically detailed integrate and fire neural networks carried out by Evans and Stringer (2012). Furthermore, the trace learning rule can be implemented in the afferent synaptic connections to all neuronal layers in the network, which avoids the biologically implausible need for separate layers for template learning and invariance learning as has been implemented in previous models. Another important factor that underpins the biological plausibility of the simulations carried out in this paper is that the network model was always trained on whole objects rather than carefully pre-segmented and isolated parts of objects corresponding to local boundary elements. Indeed, in Study 3, VisNet was trained on a random assortment of whole natural visual objects. Nevertheless, the network was still able to develop neurons that were specifically tuned to localized boundary segments of objects. We also found the performance of the model to be extremely robust, which gives additional credence to the learning mechanisms explored in this paper.

### 4.1. Future work

The version of the VisNet architecture used in this paper incorporated associative learning only in the bottom-up (feed-forward) connections between successive layers of the network. Furthermore, no top-down connections were included in the model even though these are known to exist in the primate ventral visual pathway. The rationale for using this simplified architecture in the current study was that it is sufficient to replicate how neurons in V4, TEO, and posterior TE are able to learn to encode the conformation of boundary contour elements at a particular position within an object. However, Zhou et al. (2000) have shown that the responses of neurons in earlier stages of visual processing such as V1 and V2, which have preferred responses to oriented edges, are also modulated by which side of a figure the edge occurs on. This is the case even when the figure/background cues lie well-outside the classical receptive field of the neuron. This suggests that global image context specifying border ownership modulates the activity these neurons. This contextual information must be conveyed to these early stage visual neurons by some combination of top-down connections between layers and recurrent connections within layers.

Another question is whether the approach proposed here can be extended to 3D shape. Yamane et al. (2008) have demonstrated the existence of neurons that encode the 3D configuration of localized surface fragments defined by their conformation, orientation and position with respect to the center of mass of the object. A population of such neurons provides a distributed representation of an object's 3D shape. The response characteristics of these neurons are also invariant as the object is shifted through different locations on the retina. It will be important to evaluate if a model such as VisNet, trained using stereoscopic input, can begin to capture the partonomic structure of 3D objects. Furthermore, it will be critical to assess whether learning rules, such as trace learning, can still be used to generate translationally invariant recognition processes.

However, theorists have long posited that the visual system in fact represents complex three-dimensional shapes, such as a table or a chair, by decomposing it into volumetric parts with axial symmetry (Biederman, 1987). A recent fMRI study in humans has provided evidence for this at the level of the neuronal population, where it was found that the visual system explicitly represents the relationships between the medial axes of linked object parts (Lescroart and Biederman, 2013). Consequently, more recently, Hung et al. (2012) have investigated medial axis shape coding in the inferotemporal cortex. This work extended their studies of parts-based spatial representations to “skeletal” representations involving a configuration of volumetric parts, where each part has an axis of radial symmetry or medial axis. The three-dimensional structure of an object may then be represented by a combination of the relationships between the medial axes of the object parts as well as the conformations of the surfaces of the object parts. Hung et al. (2012) confirmed that individual neurons in IT do in fact encode a configuration of both medial axis and surface fragments. In future work, we shall investigate whether the computational learning mechanisms demonstrated in this paper may also give rise to these kinds of skeletal representations.

### Conflict of interest statement

The authors declare that the research was conducted in the absence of any commercial or financial relationships that could be construed as a potential conflict of interest.

## References

[B1] Biederman I. (1987). Recognition-by-components: a theory of human image understanding. Psychol. Rev. 94, 115–147. 10.1037/0033-295X.94.2.115 3575582

[B2] Booth M. C. Rolls E. T. (1998). View-invariant representations of familiar objects by neurons in the inferior temporal visual cortex. Cereb. cortex 8, 510–523. 10.1093/cercor/8.6.510 9758214

[B3] Brincat S. L. Connor C. E. (2004). Underlying principles of visual shape selectivity in posterior inferotemporal cortex. Nat. Neurosci. 7, 880–886. 10.1038/nn1278 15235606

[B4] Cadieu C. Kouh M. Pasupathy A. Connor C. E. Riesenhuber M. Poggio T. (2007). A model of v4 shape selectivity and invariance. J. Neurophysiol. 98, 1733–1750. 10.1152/jn.01265.2006 17596412

[B5] Cox D. D. Meier P. Oertelt N. DiCarlo J. J. (2005). ‘Breaking’ position-invariant object recognition. Nat. Neurosci. 8, 1145–1147. 10.1038/nn1519 16116453

[B6] Cumming B. G. Parker A. J. (1999). Binocular neurons in v1 of awake monkeys are selective for absolute, not relative, disparity. J. Neurosci. 19, 5602–5618. 10377367 10.1523/JNEUROSCI.19-13-05602.1999 PMC6782336

[B7] Daugman J. G. (1985). Uncertainty relation for resolution in space, spatial frequency, and orientation optimized by two-dimensional visual cortical filters. J. Opt. Soc. Am. A 2, 1160–1169. 10.1364/JOSAA.2.001160 4020513

[B8] Evans B. D. Stringer S. M. (2012). Transformation-invariant visual representations in self-organizing spiking neural networks. Front. Comput. Neurosci. 6:46 10.3389/fncom.2012.00046 PMC3404434 22848199

[B9] Findlay J. M. Gilchrist I. D. (2003). Natural scenes and activities, in Active Vision: The Psychology of Looking and Seeing, Vol. 26 (Oxford: Oxford University Press), 129–150. 10.1093/acprof:oso/9780198524793.001.0001

[B10] Foldiak P. (1991). Learning invariance from transformation sequences. Neural Comput. 3, 194–200. 10.1162/neco.1991.3.2.194 31167302

[B11] Freeman J. Simoncelli E. P. (2011). Metamers of the ventral stream. Nat. Neurosci. 14, 1195–1201. 10.1038/nn.2889 21841776 PMC3164938

[B12] Giersch A. (2001). The effects of lorazepam on visual integration processes: how useful for neuroscientists? Vis. Cogn. 8, 549–563. 10.1080/13506280143000115

[B13] Hansen K. A. Kay K. N. Gallant J. L. (2007). Topographic organization in and near human visual area v4. J. Neurosci. 27, 11896–11911. 10.1523/JNEUROSCI.2991-07.2007 17978030 PMC6673353

[B14] Hubel D. H. Wiesel T. N. (1962). Receptive fields, binocular interaction and functional architecture in the cat's visual cortex. J. Physiol. 160, 106–154.2. 10.1113/jphysiol.1962.sp006837 14449617 PMC1359523

[B15] Hung C. C. Carlson E. T. Connor C. E. (2012). Medial axis shape coding in macaque inferotemporal cortex. Neuron 74, 1099–1113. 10.1016/j.neuron.2012.04.029 22726839 PMC3398814

[B16] Hung C. P. Kreiman G. Poggio T. DiCarlo J. J. (2005). Fast readout of object identity from macaque inferior temporal cortex. Science 310, 863–866. 10.1126/science.1117593 16272124

[B17] Isik L. Leibo J. Z. Poggio T. (2012). Learning and disrupting invariance in visual recognition with a temporal association rule. Front. Comput. Neurosci. 6:37. 10.3389/fncom.2012.00037 22754523 PMC3385587

[B18] Jones J. P. Palmer L. A. (1987). The two-dimensional spatial structure of simple receptive fields in cat striate cortex. J. Neurophysiol. 58, 1187–1211. 3437330 10.1152/jn.1987.58.6.1187

[B19] Kobatake E. Tanaka K. (1994). Neuronal selectivities to complex object features in the ventral visual pathway of the macaque cerebral cortex. J. Neurophysiol. 71, 856–867. 8201425 10.1152/jn.1994.71.3.856

[B20] Kohonen T. (1982). Self-organized formation of topologically correct feature maps. Biol. Cybern. 43, 59–69. 10.1007/BF00337288

[B21] Kohonen T. (2000). Self-Organizing Maps, 3rd Edn. New York, NY: Springer.

[B22] Larsson J. Heeger D. J. (2006). Two retinotopic visual areas in human lateral occipital cortex. J. Neurosci. 26, 13128–13142. 10.1523/JNEUROSCI.1657-06.2006 17182764 PMC1904390

[B23] Lescroart M. D. Biederman I. (2013). Cortical representation of medial axis structure. Cereb. Cortex 23, 629–637. 10.1093/cercor/bhs046 22387761

[B24] Li N. DiCarlo J. J. (2008). Unsupervised natural experience rapidly alters invariant object representation in visual cortex. Science 321, 1502–1507. 10.1126/science.1160028 18787171 PMC3307055

[B25] Mumford D. (1992). On the computational architecture of the neocortex. Biol. Cybern. 66, 241–251. 10.1007/BF00198477 1540675

[B26] Olshausen B. A. Anderson C. H. Van Essen D. C. (1993). A neurobiological model of visual attention and invariant pattern recognition based on dynamic routing of information. J. Neurosci. 13, 4700–4719. 8229193 10.1523/JNEUROSCI.13-11-04700.1993 PMC6576339

[B27] Pasupathy A. (2006). Neural basis of shape representation in the primate brain. Progr. Brain Res. 154, 293–313. 10.1016/S0079-6123(06)54016-6 17010719

[B28] Pasupathy A. Connor C. E. (2001). Shape representation in area v4: position-specific tuning for boundary conformation. J. Neurophysiol. 86, 2505–2519. Available online at: http://jn.physiology.org/content/86/5/2505.article-info 11698538 10.1152/jn.2001.86.5.2505

[B29] Pasupathy A. Connor C. E. (2002). Population coding of shape in area v4. Nat. Neurosci. 5, 1332–1338. 10.1038/972 12426571

[B30] Perrett D. I. Hietanen J. K. Oram M. W. Benson P. J. (1992). Organization and functions of cells responsive to faces in the temporal cortex. Philos. Trans. R. Soc. Lond. B Biol. Sci. 335, 23–30. 10.1098/rstb.1992.0003 1348133

[B31] Perrett D. I. Oram M. W. (1993). Neurophysiology of shape processing. Image Vis. Comput. 11, 317–333. 10.1016/0262-8856(93)90011-5

[B32] Perrett D. I. Rolls E. T. Caan W. (1982). Visual neurones responsive to faces in the monkey temporal cortex. Exp. Brain Res. 47, 329–342. 10.1007/BF00239352 7128705

[B33] Petkov N. Kruizinga P. (1997). Computational models of visual neurons specialised in the detection of periodic and aperiodic oriented visual stimuli: bar and grating cells. Biol. Cybern. 76, 83–96. 10.1007/s004220050323 9116079

[B34] Pettet M. W. Gilbert C. D. (1992). Dynamic changes in receptive-field size in cat primary visual cortex. Proc. Natl. Acad. Sci. U.S.A. 89, 8366–8370. 10.1073/pnas.89.17.8366 1518870 PMC49919

[B35] Riesenhuber M. Poggio T. (1999). Hierarchical models of object recognition in cortex. Nat. Neurosci. 2, 1019–1025. 10.1038/14819 10526343

[B36] Rodríguez-Sánchez A. J. Tsotsos J. K. (2012). The roles of endstopped and curvature tuned computations in a hierarchical representation of 2D shape. PLoS ONE 7:e42058. 10.1371/journal.pone.0042058 22912683 PMC3415424

[B37] Rolls E. T. (2000). Functions of the primate temporal lobe cortical visual areas in invariant visual object and face recognition. Neuron 27, 205–218. 10.1016/S0896-6273(00)00030-1 10985342

[B38] Rolls E. T. Cowey A. Bruce V. (1992). Neurophysiological mechanisms underlying face processing within and beyond the temporal cortical visual areas [and discussion]. Philos. Trans. Biol. Sci. 335, 11–21. 10.1098/rstb.1992.0002 1348130

[B39] Rolls E. T. Deco G. (2002). Computational Neuroscience of Vision, 1st Edn. Oxford: Oxford University Press.

[B40] Rolls E. T. Milward T. (2000). A model of invariant object recognition in the visual system: learning rules, activation functions, lateral inhibition, and information-based performance measures. Neural Comput. 12, 2547–2572. 10.1162/089976600300014845 11110127

[B41] Rolls E. T. Treves A. (1998). Neural Networks and Brain Function, 1st Edn. Oxford: Oxford University Press.

[B42] Rolls E. T. Treves A. Tovee M. J. Panzeri S. (1997). Information in the neuronal representation of individual stimuli in the primate temporal visual cortex. J. Comput. Neurosci. 4, 309–333. 10.1023/A:1008899916425 9427118

[B43] Rumelhart D. E. Zipser D. (1985). Feature discovery by competitive learning^*^. Cogn. Sci. 9, 75–112. 10.1207/s15516709cog0901.512662852

[B44] Serre T. Kouh M. Cadieu C. Knoblich U. Kreiman G. Poggio T. (2005). A Theory of Object Recognition: Computations and Circuits in the Feedforward Path of the Ventral Stream in Primate Visual Cortex. Cambridge, MA: MIT CSAIL.

[B45] Serre T. Oliva A. Poggio T. (2007). A feedforward architecture accounts for rapid categorization. Proc. Natl. Acad. Sci. U.S.A. 104, 6424–6429. 10.1073/pnas.0700622104 17404214 PMC1847457

[B46] Silver M. A. Kastner S. (2009). Topographic maps in human frontal and parietal cortex. Trends in Cogn. Sci. 13, 488–495. 10.1016/j.tics.2009.08.005 19758835 PMC2767426

[B47] Stringer S. M. Perry G. Rolls E. T. Proske J. H. (2006). Learning invariant object recognition in the visual system with continuous transformations. Biol. Cybern. 94, 128–142. 10.1007/s00422-005-0030-z 16369795

[B48] Stringer S. M. Rolls E. T. (2008). Learning transform invariant object recognition in the visual system with multiple stimuli present during training. Neural Netw. 21, 888–903. 10.1016/j.neunet.2007.11.004 18440774

[B49] Stringer S. M. Rolls E. T. Tromans J. M. (2007). Invariant object recognition with trace learning and multiple stimuli present during training. Network 18, 161–187. 10.1080/09548980701556055 17966074

[B50] Tanaka K. Saito H. Fukada Y. Moriya M. (1991). Coding visual images of objects in the inferotemporal cortex of the macaque monkey. J. Neurophysiol. 66, 170–189. 1919665 10.1152/jn.1991.66.1.170

[B51] Tromans J. M. Harris M. Stringer S. M. (2011). A computational model of the development of separate representations of facial identity and expression in the primate visual system. PLoS ONE 6:e25616. 10.1371/journal.pone.0025616 21998673 PMC3188551

[B52] Tsao D. Y. Freiwald W. A. Knutsen T. A. Mandeville J. B. Tootell R. B. H. (2003). Faces and objects in macaque cerebral cortex. Nat. Neurosci. 6, 989–995. 10.1038/nn1111 12925854 PMC8117179

[B53] Tsotsos J. K. (1993). An inhibitory beam for attentional selection, in Proceedings of the 1991 York Conference on Spacial Vision in Humans and Robots, (New York, NY: Cambridge University Press), 313–331.

[B54] Tsunoda K. Yamane Y. Nishizaki M. Tanifuji M. (2001). Complex objects are represented in macaque inferotemporal cortex by the combination of feature columns. Nat. Neurosci. 4, 832–838. 10.1038/90547 11477430

[B55] vanRullen R. (2008). The power of the feed-forward sweep. Adv. Cogn. Psychol. 3, 167–176. 10.2478/v10053-008-0022-3 20517506 PMC2864977

[B56] von der Malsburg C. (1973). Self-organization of orientation sensitive cells in the striate cortex. Kybernetik 14, 85–100. 4786750 10.1007/BF00288907

[B57] Wallis G. (2013). Toward a unified model of face and object recognition in the human visual system. Front. Psychol. 4:497 10.3389/fpsyg.2013.00497 PMC3744012 23966963

[B58] Wallis G. Rolls E. T. (1997). Invariant face and object recognition in the visual system. Progr. Neurobiol. 51, 167–194. 10.1016/S0301-0082(96)00054-8 9247963

[B59] Yamane Y. Carlson E. T. Bowman K. C. Wang Z. Connor C. E. (2008). A neural code for three-dimensional object shape in macaque inferotemporal cortex. Nat. Neurosci. 11, 1352–1360. 10.1038/nn.2202 18836443 PMC2725445

[B60] Yarbus A. L. (1967). Eye Movements During Perception of Complex Objects. New York, NY: Springer US.

[B61] Zhou H. Friedman H. S. von der Heydt R. (2000). Coding of border ownership in monkey visual cortex. J. Neurosci. 20, 6594–6611. 10964965 10.1523/JNEUROSCI.20-17-06594.2000 PMC4784717

